# Unraveling the Most Relevant Features for the Design of Iridium Mixed Oxides with High Activity and Durability for the Oxygen Evolution Reaction in Acidic Media

**DOI:** 10.1021/jacsau.3c00247

**Published:** 2023-08-23

**Authors:** Dmitry Galyamin, Álvaro Tolosana-Moranchel, María Retuerto, Sergio Rojas

**Affiliations:** Grupo de Energía y Química Sostenibles. Instituto de Catálisis y Petroleoquímica, CSIC, C/Marie Curie 2, 28049 Madrid, Spain

**Keywords:** Water Electrolysis, Green-Hydrogen, Iridium, Mixed-Oxide, Oxygen Evolution Reaction, Connectivity, PEMWE

## Abstract

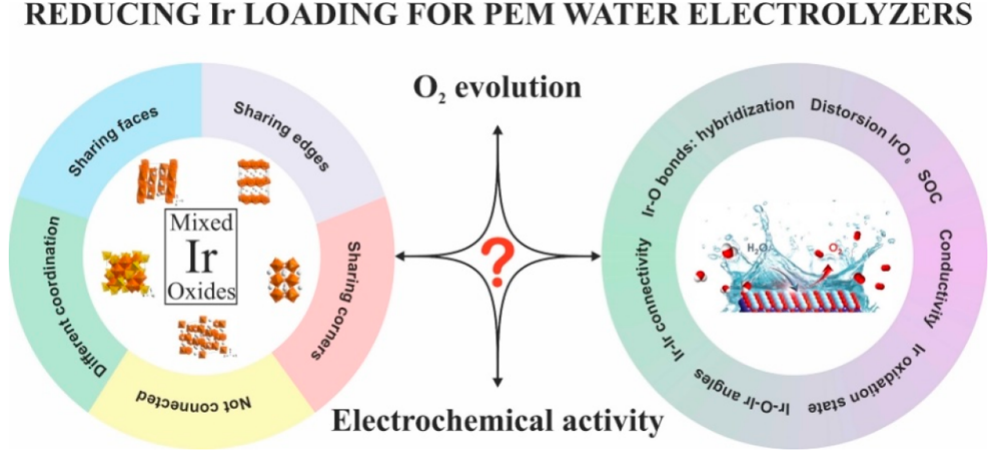

Proton exchange membrane water electrolysis (PEMWE) is the technology of choice for the large-scale production of green hydrogen from renewable energy. Current PEMWEs utilize large amounts of critical raw materials such as iridium and platinum in the anode and cathode electrodes, respectively. In addition to its high cost, the use of Ir-based catalysts may represent a critical bottleneck for the large-scale production of PEM electrolyzers since iridium is a very expensive, scarce, and ill-distributed element. Replacing iridium from PEM anodes is a challenging matter since Ir-oxides are the only materials with sufficient stability under the highly oxidant environment of the anode reaction. One of the current strategies aiming to reduce Ir content is the design of advanced Ir-mixed oxides, in which the introduction of cations in different crystallographic sites can help to engineer the Ir active sites with certain characteristics, that is, environment, coordination, distances, oxidation state, etc. This strategy comes with its own problems, since most mixed oxides lack stability during the OER in acidic electrolyte, suffering severe structural reconstruction, which may lead to surfaces with catalytic activity and durability different from that of the original mixed oxide. Only after understanding such a reconstruction process would it be possible to design durable and stable Ir-based catalysts for the OER. In this Perspective, we highlight the most successful strategies to design Ir mixed oxides for the OER in acidic electrolyte and discuss the most promising lines of evolution in the field.

## Introduction

Despite the increasing number of government initiatives, plans, and actions to limit the temperature rise to 1.5 °C,^1−3^ global CO_2_ emissions have increased every year since 2015, with the exception of 2020. Electrification of energy and production sectors with renewable energy and the use of green hydrogen and sustainable biomass in sectors where direct electrification is not feasible are the cornerstones for meeting the 1.5 °C target.^4^ In addition, and triggered by recent geopolitical tensions, many countries have taken decisive actions to bolster their energy security mostly by promoting the use of domestically produced renewable energy. Within this context of energy transition, (green) hydrogen is gaining momentum in both academia and industry.^5^ The term green hydrogen was coined to identify the hydrogen obtained from carbon-decoupled sources, ideally by using renewable energy.^6^ It can be produced by several technologies, including water electrolysis, biomass gasification, and photochemical and thermochemical water splitting.^7^

Water electrolysis is the most developed technology for producing green hydrogen in the GW to TW scale needed to realize the climate neutral targets.^6^ There are several types of electrolyzers, namely, alkaline electrolyzers (AWE), Proton Exchange Membrane Water Electrolyzer (PEMWE), Anion Exchange Membrane Water electrolyzer (AEMWE), oxygen ion-conducting Solid Oxide Electrolyzer (SOEC), and proton-conducting SOEC, also called proton-conducting ceramic electrolyzer (PCCEL), with the first two technologies being the most developed ones.^8^

Electrolyzers can be classified according to different operational or composition parameters. Thus, AWE, AEMWE, and PEMWE operate at low temperatures (40–90 °C) while solid oxide technologies work above 300 °C in the case of PCCEL and around 700 °C for SOEC.^5^ On the other hand, in AWE and AEMWE, hydroxyl ions (OH^–^) are transported through the electrolyte, namely, a diaphragm in AWE or a membrane in AEMWE, whereas protons (H^+^) are transported in PEMWE and PCCEL. AWE and PEM electrolyzers are already commercially available and in fact alkaline electrolyzers are widely used in the chlori-alkaline industry to produce chlorine and sodium hydroxide, but also hydrogen.^5,8^ The components of AEM electrolyzers are based upon Ni-based materials, which is a clear benefit to their scaling-up. The main drawback of AWE is the intermixing between the H_2_ and O_2_ generated during operation, and work at low operating pressures. In addition, they are optimized for continuous operation, a feature that conflicts with the intermittent production of renewable electricity. These features can be avoided with AEMWE, since they can operate at high pressure, while obtaining high purity hydrogen without using PGMs in their components. However, the membranes still need to be improved with mechanical and thermal stability in the electrolysis conditions, efficient to OH^–^ transfer, low permeability of gases, etc.^5,7,9^ PEMWE technology is the most effective technology for the production of green hydrogen from renewable electricity. PEMWE uses solid electrolyte membranes based upon perfluorinated sulfonic acid copolymer membranes, which display high ionic conductivity (low resistivity 5–6 Ω cm at 80–90 °C), resulting in high efficiencies and allowing the possibility of working at high current densities (4–6 A cm^–2^ or even higher).^5,10^ PEMWE can work in a dynamic mode, with a very rapid transition between idle and full load periods (in less than one s at nominal temperature). As a consequence, they are suitable for work under the dynamic operation conditions imposed by the intermittency of renewable electricity generation. Moreover, due to the compact design, lower thermal capacity and operating temperature cold start-up in scale-up PEMWE systems have been reported to take 5–10 min.^11^ Due to the low gas crossover, high-purity hydrogen (99.99–99.9999%) can be obtained, which furthermore can be pressurized during electrolysis, reducing the costs of subsequent pressurization. Finally, they have a very compact and easy-to-stack design, which allows them to be easily scaled.^12,13^ The most critical feature of PEMWE is the use of costly materials in the electrodes: Pt and Ir in the cathode and anode electrodes, respectively, and Ti in the bipolar plates and porous transport layers and the degradation of the polymeric membrane. SOEC technologies can operate at high current densities and high efficiency, and their electrochemical processes are reversible, allowing them to work in fuel cell or electrolysis mode. However, the electrochemical degradation and thermomechanical stability of their components still need to be improved.^14^ PCCELs are also very efficient, but they still have difficulties in the configuration of the electrolyte/porous electrode support and also poor thermomechanical properties.^15^

The basic operation of a PEMWE is as follows: at the anode electrode, the Oxygen Evolution Reaction (OER; H_2_O → O_2_ + 4H^+^ + 4e^–^) takes place in the presence of an Ir-based catalyst. At the cathode electrode, the Hydrogen Evolution Reaction (HER; 4H^+^ + 4e^–^ → 2H_2_) takes place. Although H_2_ is the desired product, the overall water electrolysis reaction is limited by the sluggish kinetics for the OER. As a result, most of the research concerning electrocatalyst development for water electrolysis focuses on the OER.^16−20^ Note also that the OER is a key process in other applications such as photoelectrochemical devices, regenerative cells and metal-air batteries, so the improvement of this reaction is essential in the whole scenario of sustainable energy.^21,22^

Although water electrolysis is a commercially available technology, the cost of green hydrogen is higher than that of hydrogen produced from fossil-fuels, typically steam methane reforming.^6,23^ This accounts to several features, including the high price of renewable electricity and to the high price of today’s PEMWE.^8^ This scenario is expected to change in the near term, with projected prices of renewable hydrogen close to, or below, 1 USD/kg_H2_.^24^ On the one hand, the price of renewable electricity is decreasing rapidly, mostly due to the decreasing price of photovoltaic panels. On the other hand, the high cost of today’s commercially available PEMWEs stacks accounts to the use of nonoptimized materials and components, and low-scale production.^8^ Platinized titanium bipolar plates (BPs) and porous transport layers (PTLs) are major cost components, with the cost of the catalyst coated membrane (CCM) representing around 24% of the total cost of the stack for a 1 MW PEMWE stack electrolyzer, with the contribution of PGMs (Ir and Pt) used in the electrodes representing around 5–8% of the total price.^8^ The novel generation of PEMWE are expected to contain a significantly lower amount of Platinum Group Metal (PGM) elements (Ir and Pt), while replacing Ti-based components (PTLs and BPs) by affordable stainless-steel based ones.^25^

Due to the very fast kinetics of the HER in acidic electrolyte, Pt loading can be reduced without losing performance, the typical Pt loading in the cathode being ≈0.5–1.0 mg_Pt_ cm^–2^.^26^ Ir-based electrocatalysts are the only ones that can withstand the harsh oxidizing conditions in the anode electrodes of PEMWEs. Due to the sluggish kinetics of the OER a high iridium loading (between 1 and 3 mg_Ir_ cm^–2^) is used in the anode electrode of today’s PEMWEs.^26^ Ir is one of the rarest and ill-distributed elements on earth’s crust. As a consequence, Ir supply is currently dominated by South Africa (ca. 87% of global Ir production), followed by Zimbabwe (8%), Russia and Canada (3% each).^27^ Therefore, Ir is extremely expensive (4600 $/troy oz as of May 2023).^28^ In addition, the very small market results in Ir prices being strongly subjected to speculative purchasing, hence posing a serious risk for the large scale (GW to TW) deployment of electrolyzers. It is therefore needed to develop novel anode electrodes that can display similar performances with lower Ir loadings of ca. 0.5–0.2 mg_Ir_ cm^–2^ or lower, that can deliver high current density at low potentials while displaying high durability in the range of tens of thousands of hours.

In this Perspective, we will first identify the state-of-the-art electrocatalysts for the OER in acidic media. Next, we will introduce the Ir-mixed oxides reported in the literature and analyze features such as their crystal structure and morphology and their relationship with their OER activity. Finally, we will discuss the evolution of the Ir-mixed oxide structure and composition during the OER and indicate good practices and recommendations to assess their OER performance.

## Oxygen Evolution Reaction, Mechanism, and Benchmark Ir Catalysts

Through this paper, we will report and discuss the OER activity and durability data obtained in aqueous acidic electrolyte using rotating disk electrodes (RDEs) or, when available, in lab-scale PEMWE. Although the performance of promising newly developed catalysts should be tested in PEMWE, RDE is a suitable approach for the fast screening of a large number of electrocatalysts, usually prepared in the milligram scale. In fact, most scientific studies dealing with the development of novel electrocatalysts report only activity and durability data from RDE experiments. Sadly, and contrary to the oxygen reduction reaction (ORR), a benchmark protocol for testing OER performance of an oxygen ion in RDE (and in PEMWE) has not been universally implemented and followed by the scientific community yet, so activity data obtained in a wide range of reaction conditions are usually reported. As a consequence, it is very difficult, if not simply impossible, to establish proper comparisons between the OER performances of novel electrocatalysts measured in different laboratories. The final section of this perspective will discuss both approaches and indicate recommendations to perform RDE testing.

### Mechanisms for the Oxygen Evolution Reaction in Acid Media

**Figure 1:**
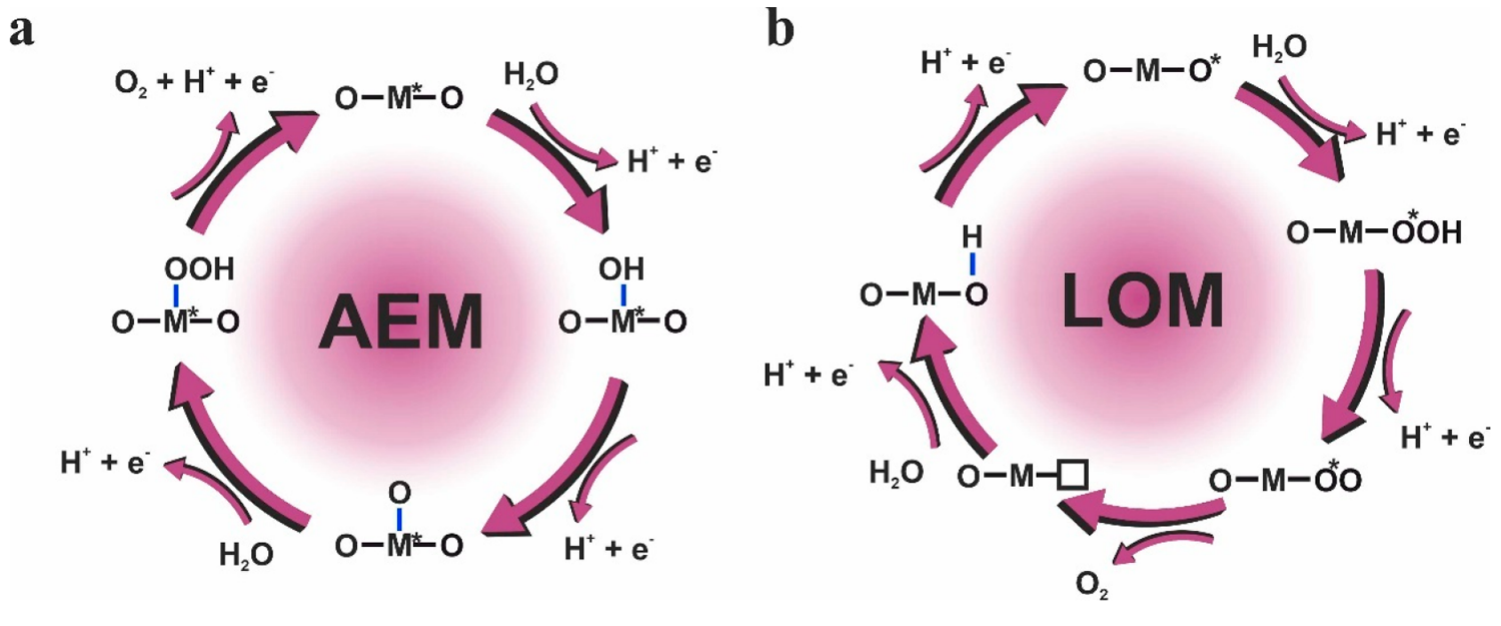
Schematic representation of (a) AEM and (b) LOM mechanisms for the OER. Adapted with permission from ref (20). Copyright 2021 MDPI.

The two most accepted mechanisms by the scientific community are the adsorbate evolution mechanism (AEM) and the lattice-oxygen mechanism (LOM). The AEM mechanism was proposed by Rossmeisl and co-workers through DFT calculations.^29^ The mechanism is schematized in Figure 1a, where the asterisk (*) represents a surface-active site. The overpotential of the OER is strongly related to O* surface binding energies of the O*, and therefore, the electrochemical activity is limited by the O* and the OOH* formation steps. For materials that bind O* too strongly, the activity is limited by the formation of OOH*, whereas for surfaces on which O* binds too weakly, it is limited by the O* formation. For example, RuO_2_ binds O* a little too weakly, whereas IrO_2_ binds it too strongly.^29^

The first two steps of the LOM mechanism (Figure 1b) are the same as those for the AEM. The difference in the following steps is that in the LOM, the O* together with an oxygen from the catalyst forms O_2_(g), in such a way that an oxygen vacancy (□) is generated on the surface. Finally, this vacancy is replaced with oxygen from a water molecule. Clearly, the presence of oxygen vacancies is a key feature in the LOM.

Unfortunately, and to the best of our knowledge, it remains unknown which structural or compositional factors make a catalyst proceed through a specific mechanism. As observed from Figure 1, the active sites for the AEM are coordinating unsaturated metal ions, whereas the active sites for the LOM are coordinating unsaturated oxygen ions. The LOM mechanism is favored when the Fermi level decreases to the oxygen 2*p* band induced by the strong overlap of metal 3*d* with oxygen 2*p*.^30^ Moreover, the LOM pathway is easier to occur when the catalyst’s lattice parameters are small and the separation between surface oxygen is short, features than can be controlled by tuning the bending of the metal–O–metal bond by properly choosing the nature of the metal.^30^ Moreover, the presence of oxygen vacancies seems to be an essential factor for the occurrence of the LOM.^17,19,31^ Thus, it is more likely to observe the LOM process for amorphous catalysts since it is easier to add or remove oxygen in the lattice. For more information about the OER mechanisms, please refer to the following references: refs (32) and (33).

### Benchmark OER Iridium-Based Catalysts

The benchmark electrocatalyst for the OER in acidic media is rutile IrO_2_. However, some papers report activity values obtained with homemade IrO_2_,^34,35^ while other researchers use commercial IrO_2_ samples obtained from different suppliers such as Umicore, Haereus, Sigma-Aldrich Corporation, or Alfa Aesar.^36,37^ For instance, Ir mass-normalized activities of ca. 10 and 42 A g_Ir_^–1^ at 1.525 and 1.6 V, respectively, were obtained with IrO_2_ purchased from Sigma-Aldrich (IrO_2_, 99.9%) in 0.5 M H_2_SO_4_.^36^ However, mass-normalized activities of 50 and 475 A g_Ir_^–1^ at 1.525 and 1.6 V, respectively, were obtained with an IrO_2_ catalysts purchased from Alfa Aesar (99% Ir).^36^ In agreement with the former value, a similar value of ca. 30 A g_Ir_^–1^ at 1.51 V was reported by Daiane Ferreira da Silva et al.^38^ for an IrO_2_ catalyst purchased from Alfa Aesar (99.99% trace metals basis) in 0.05 M H_2_SO_4_. Moreover, they tested several supported and unsupported Ir-based catalysts for the OER and concluded that whereas supported samples provided higher activity than unsupported ones, they also showed less stability. As shown above, the activity of the Ir-based electrocatalysts strongly depends on the nature of the initial Ir species, the synthesis procedure and the protocol for the OER measurement.^36,39^ Therefore, it is highly recommended to compare the activity results to those obtained by using commercially available electrocatalysts.

In addition, the activity of IrO_2_ measured in PEMWE depends on a number of factors,^40^ including, but not only, the thickness of the membrane, the structure of the PTL and its coating, the cell temperature, and the catalyst loading on the electrode. A catalyst coated membrane (CCM) produced with Nafion 212 and an Ir loading of 2 mg_Ir_ cm^–2^ on the anode at a temperature of 80 °C can reach a current density of 4 A cm^–2^ at 1.79 V (70% efficiency vs LHV).^41^ When measured in PEMWE, Ir-mass normalized activities of 12 and 420 A g_Ir_^–1^ have been reported with two different catalysts, namely, c-IrO_2_/TiO_2_ purchased from Umicore and a-IrO(OH)_*x*_/TiO_2_ from Heraeus Deutschland, respectively.^42^ Contrary to RDE measurements, when measured in PEMWE cell, Ir-based electrocatalyst are usually supported/dispersed onto a (more or less) conducting oxide, typically TiO_2_, or due to its poor conductivity, onto ATO (antimony tin oxide),^12,26,43,44^ Due to the low conductivity of these oxides, a high loading of Ir, between 40 and 70 wt %, is usually needed to produce a catalyst layer with sufficient conductivity.

In order to succeed in the realization of a hydrogen economy based on water electrolyzers in the GW to TW scale, it is imperative to decrease Ir loading on the electrodes of PEMWEs from today’s 1–3 mg_Ir_ cm^–2^ to more feasible values of around 0.2–0.4 mg_Ir_ cm^–2^, or lower. However, several studies concluded that decreasing Ir loading to 0.5 mg_Ir_ cm^–2^ using the same IrO_2_/TiO_2_ catalysts that are used in state-of-the-art CCMs with 2 mg_Ir_ cm^–2^, will result in very thin and inhomogeneous catalyst layers resulting in high performance loss.^16^ Therefore, novel catalysts should be designed to be used in low loaded CCMs. These catalysts should display high mass-normalized OER and durability but also display high conductivity and optimized packing density.

## Strategies to Increase Ir’s Activity for the OER

The most traditional approach to increase Ir mass-normalized activity is to maximize catalyst dispersion, i.e., to increase the fraction of surface atoms vs bulk atoms. One obvious strategy to maximize catalyst dispersion is to reduce the size of Ir particles to the nanometer range. Reier et al.^45^ reported that Ir nanoparticles have comparable OER activity and durability to bulk Ir, indicating their potential as nanoscaled OER catalyst. However, the electrochemical surface characteristics of Ir nanoparticles differed from those of bulk Ir material, with Ir nanoparticles losing their voltammetric metallic features during voltage cycling, indicating changes in oxidation chemistry. Overall, the results suggest that Ir nanoparticles could be a promising option for the OER catalysis. A number of studies have focused on synthesizing nanoscale IrOx materials to improve their mass activity compared to bulk IrO_*x*_.^46^ These studies include rutile nanoparticles, Ir nanodendrites^47^ or Ir single atoms.^48^ Results showed potentials of around 1.48 V for Ir single atoms and 1.65 V for Ir nanoparticles in the RDE at 10 mA cm^–2^. Ir nanoparticles were also studied in a MEA type electrolyzer^49^ and displayed a 10-fold higher OER activity compared to that of flake-like structured Ir-black, not only because of the increase in the surface area but also due to the nanoporous structure of the Ir-nano catalyst. These findings suggest that nanoscale IrO_*x*_ materials have the potential to improve the efficiency of the OER catalysis. Recently, the formation of IrO_2_ nanoribbons, with the monoclinic phase, has been reported to result in very active Ir sites for the OER with a low potential of 1.435 V to achieve 10 mA cm^–2^ in RDE.^50^ Other approaches for increasing the fraction of Ir atoms at the surface include the formation of core@shell structures, in which core elements, which are usually inactive particles such as Au,^51^ Co,^52^ or Pd.^53^

Theoretical studies suggest that the energy of oxygen bonding onto the surface of the catalyst determines its OER activity, with rutile RuO_2_ and IrO_2_ displaying the most favorable energies. However, neither of them is located at the apex of the volcano curve; RuO_2_ binds oxygen too weak and IrO_2_ too strong.^29^ In principle, it would be possible to tune the energy of oxygen adsorption on the catalyst surface by doping RuO_2_ or IrO_2_ with other metals. The number of doping agents is very high, including transition metals such as Ti, Mn, Mo, etc., or noble metals such as Pd, Au, or Ru.^54−56^ For instance, RuIrO_*x*_ improved the activity of IrO_2_, yielding 1225 A g^–1^_noble-metal_.^57^ One of the most active catalysts was obtained by doping IrO_2_ with Ta and Tm by using a fast pyrolysis, resulting in an mass-normalized activity of 3126 A g^–1^_Ir_ at 1.5 V for Ta_0.1_Tm_0.1_Ir_0.8_O_*x*_.^58^ Other catalysts based on doped IrO_*x*_ showing high mass-normalized activity have been reported: 100 and 900 A g_Ir_^–1^ for Li–IrO_*x*_ and W_0.8_Ir_0.2_O_*y*_, respectively, at 1.525 V.^59−61^ The nature of the doping agent can affect parameters such as Ir’s oxidation state, Ir–Ir and Ir–O–Ir bond lengths, and orbital hybridization. The increased activity of Ta_0.1_Tm_0.1_Ir_0.8_O_*x*_ was ascribed to strain and tuned electronic structure leading to an optimized electronic structure. Lower oxidation state than +4 and longer length of the first shell Ir–O (2.06 Å) compared to IrO_*x*_ were also observed. The improved activities of Ir_0.6_Mn_0.4_O_*x*_ and RuIrO_*x*_ were ascribed to the existence of Ir^3+^ and a shift of the Fermi level to the *d* band center. Consequently, the adsorption of oxygen intermediates is enhanced and the energy barrier of the potential-determining step decreased.^57,61^ Other effect that has been reported to result in higher OER activity is the segregation of phases, giving rise to the formation of iridium oxide on the surface.^60^ However, when IrO_*x*_ was doped with Li the faster OER was ascribed to the formation of more flexible, disordered IrO_6_ octahedra, which are more easily oxidized during OER, along with the shrinkage of the Ir–O bond, acting as more electrophilic centers.^59^

In doped-IrO_*x*_ (or Ir black), the dopant element replaces a fraction of Ir cations in the IrO_*x*_ network, with both atoms occupying the same crystallographic positions randomly. Therefore, it is difficult, if not impossible, to pinpoint the precise location of the dopant atoms in the oxide network, hence to understand or tune the effect of such element. In the following sections we will present Ir-mixed oxides as potential candidates to develop advanced OER catalysts with high activity and durability. Due to their unique characteristics, namely, easy control of their composition and structure, Ir mixed-oxides allow the identification of accurate structure–activity descriptions, that would permit one to rationalize the designing of advanced Ir catalysts.

## Iridium Mixed Oxides

Iridium mixed oxides are usually called iridates. A mixed oxide can be defined as an oxide with different cations in different, well-defined crystallographic positions. Mixed oxides are very versatile in terms of composition, crystal structure, distortions, electronic configurations, different possible environments for the cations, and Ir’s oxidation state. In addition, due to the wide range of elements with different oxidation states that can be incorporated, it is likely that mixed oxides can present oxygen vacancies and mixed cationic valences. Ir-mixed oxides display two main advantages as electrocatalysts for the OER. First, the content of Ir per unit formula is reduced, and second since the location and content of M (hence of Ir) can be controlled and determined accurately, the actual environment/coordination of Ir can be tuned precisely.

In the next sections, we will identify the Ir-mixed oxides reported in the literature and group them according to their environment and type of connection between the IrO_*x*_ units. We will report the OER activity of selected Ir-mixed oxides in terms of current density and, when possible, Ir mass-normalized activity. This will allow us to identify the most relevant parameters that affect the OER activity of Ir-mixed oxides, and will allow us to propose further Ir-mixed oxides worth to be studied as electrocatalyst for the OER. Finally, we will address the dissolution/reconstruction of Ir-mixed oxides and its repercussions for the OER.

### Iridium-Mixed Oxides, Crystallographic Structures, and OER Activity

**Figure 2:**
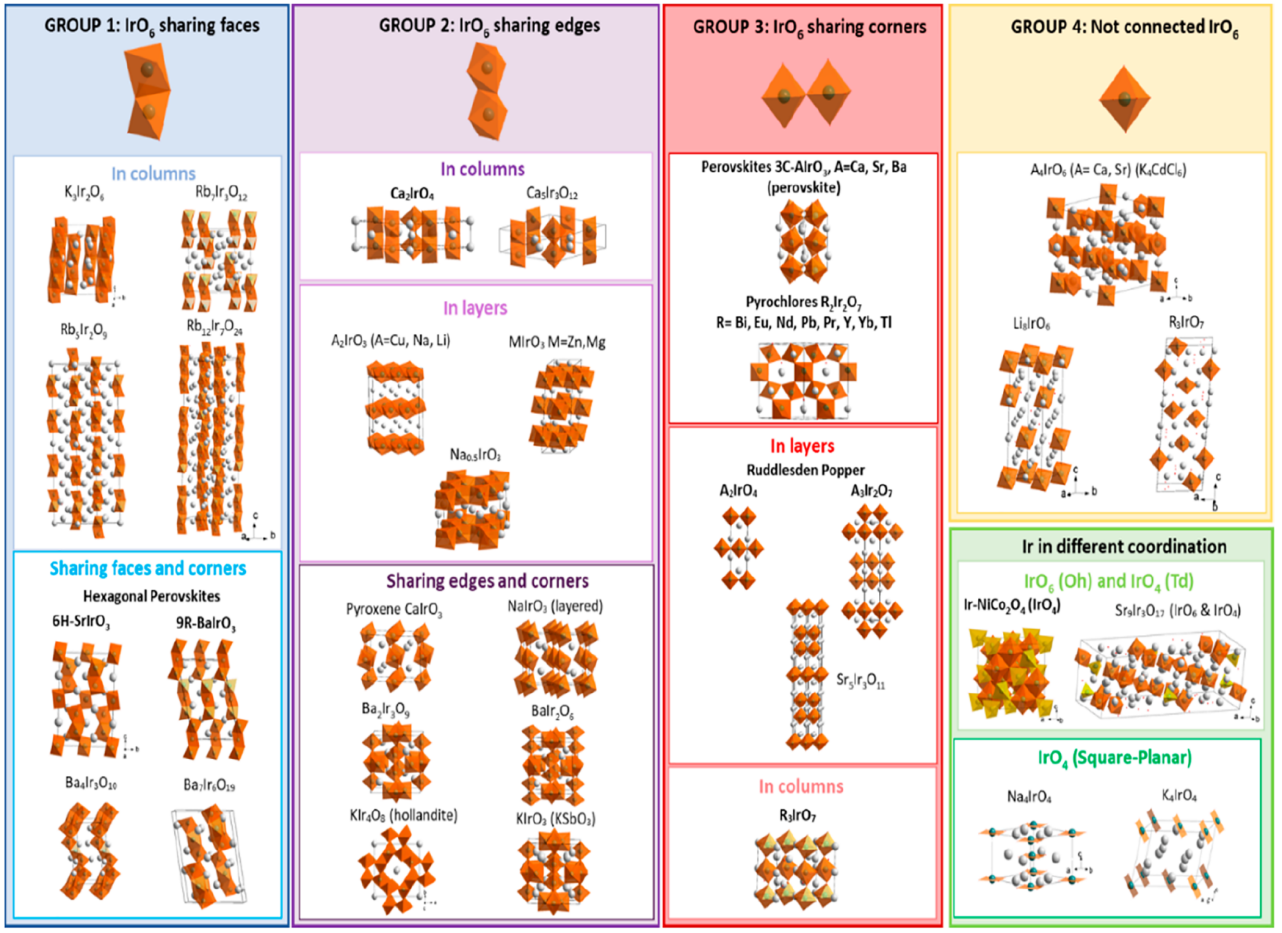
Crystallographic structures of the most typical Ir-mixed oxides. IrO_6_ octahedra are represented in orange, IrO_4_ in tetrahedra in yellow, and IrO_4_ in square-planar in light orange. The compounds identified in bold have been studied as electrocatalysts for the OER.

Figure 2 depicts the different crystallographic structures reported for Ir-mixed oxides. Note that Figure 2 shows the structure of the paradigmatic material of each system, although many modifications are possible by introducing other cations into the structures. To date, only some of the mixed oxides depicted in Figure 2 have been studied as electrocatalysts for the OER. Table S1 and Figures S1 and S2 report a comprehensive collection of OER activity data for the Ir-mixed oxides reported in the literature. With the aim of unveiling the most relevant parameters that influence the OER activity, the mixed-oxides shown in Figure 2 are collated according to geometric considerations, in particular, the interconnection between the IrO_*x*_ units that conform the oxide. When appropriate, features other than geometry will be also considered during the discussion of the OER activity.

As observed in Figure 2, Ir-mixed oxides are usually arranged in a network of Ir cations occupying octahedral (Oh) sites, surrounded by 6 oxygen atoms (IrO_6_). These octahedral units are the basic building blocks of the crystal structure of Ir-mixed oxides. The way these octahedra are connected defines their structure. Thus, IrO_6_ octahedra can be isolated (displaying no connection between them), share corners, share edges or share faces. In some cases, IrO_6_ can display more than one type of connection and share, for instance, corners and faces. The connection between the IrO_6_ in the mixed-oxide network determines features such as the Ir–Ir and Ir–O distances, Ir–O–Ir angles, distortions, etc. As a result, the extension of orbital interactions between the Ir and oxygen ions is affected, hence determining parameters such as the conductivity, the electronic band structure and the spin–orbit coupling, features that inform OER performance.^62,63^ For the sake of discussion, we have considered four groups of iridates attending to the connection between their IrO_6_ units:

### Group 1 Iridates

**Figure 3:**
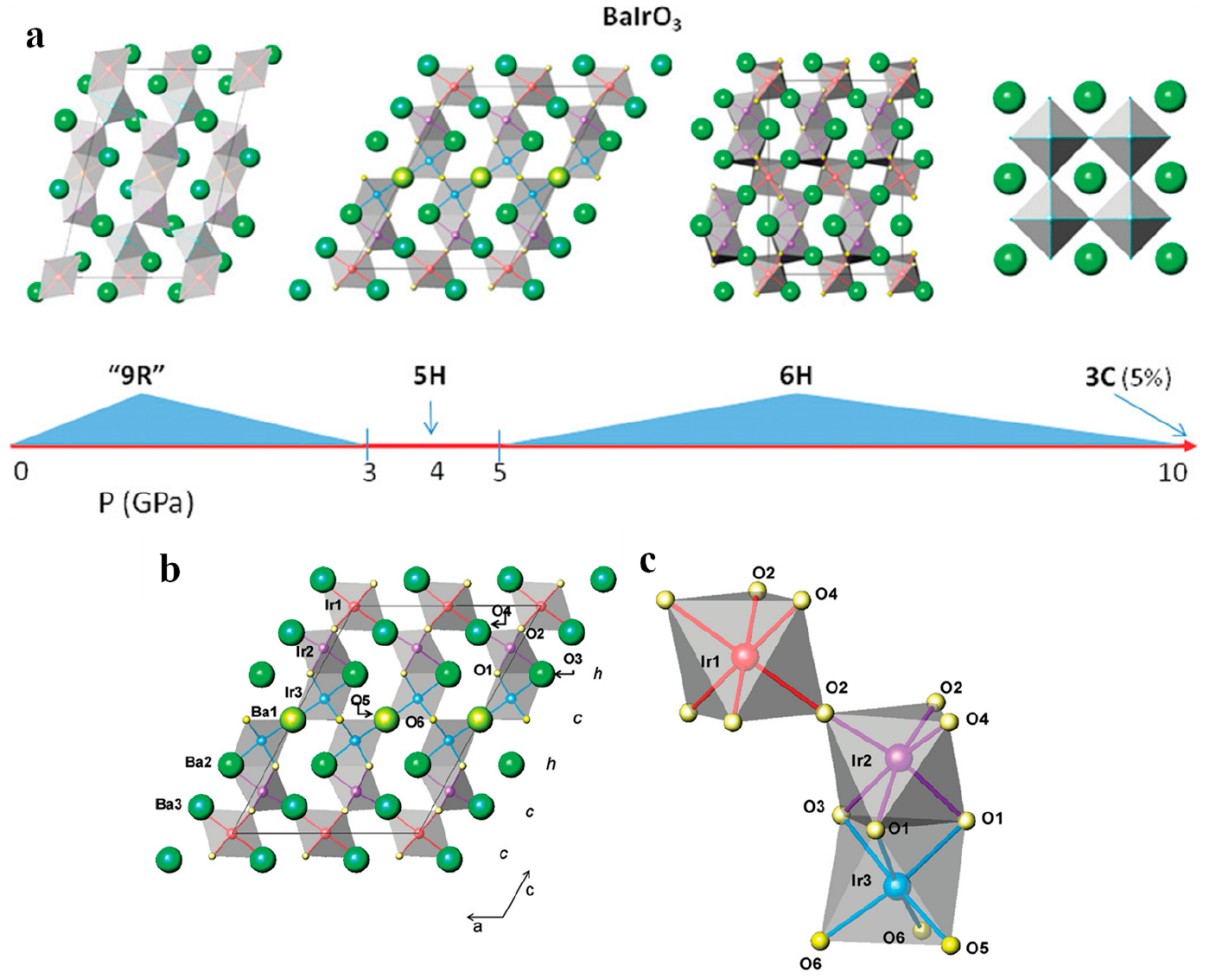
(a) Different polytypes of BaIrO_3_ depending on the synthesis conditions. (b) Example of the crystal structure of the 5H polytype along [0 1 0], showing the stacking of hexagonal (h) and cubic (c) layers corresponding to face-sharing or corner-sharing IrO_6_ octahedra, respectively. (c) Close look at the dimer and corner-shared octahedra. Figure reproduced from ref (65). Copyright 2009 American Chemical Society.

This group contains iridates with IrO_6_ connected through the faces, and it can be separated into two subgroups:

(i) Compounds arranged in isolated columns of face-sharing IrO_6_ octahedra, that can be dimers (Ir_2_O_9_), trimers (Ir_3_O_12_), or longer units, such as K_3_Ir_2_O_6_, which can be considered as a quasi-1D system.^64^ To the best of our knowledge, none of these materials has been studied for the OER.

(ii) Compounds based on face- and corner-shared IrO_6_ octahedra. These compounds also form dimers or trimers of IrO_6_ sharing faces in a two-dimensional zigzag, connected between them through corners. These compounds are usually referred to as hexagonal perovskites. In the ABO_3_ perovskites, the Goldschmidt tolerance factor, defined as  where *r*_A_ and *r*_B_ are the ionic radii of the A and B site cations, respectively, and *r*_O_ is the ionic radius of the oxygen ion, determines the structure of the perovskite. When *t* = 1, the ideal perovskite structure, i.e., a three-dimensional framework built up by corner-sharing BO_6_ octahedra, is stabilized (see below). Hexagonal perovskites have a tolerance factor *t* > 1. This is because A cations are too large compared to B cations to stabilize the ideal perovskite, resulting in the face-sharing connection. In general, the IrO_6_ octahedra in these structures are highly distorted. Several hexagonal stackings, 6H, 5H, and 9R, can be formed (see Figure 3). For instance, in 6H-SrIrO_3_, face-sharing IrO_6_ dimers are connected by corners to single IrO_6_ octahedra. Figure 3 illustrates the different polymorphs for BaIrO_3_ that can be obtained depending on the synthesis conditions.

In general, oxides from this subgroup display a very high OER activity and durability. For instance, the OER activity of 6H-SrIrO_3_ is 1.478 V at 10 mA cm^–2^, or 75 Ag^1–^_Ir_ at 1.525 V. This catalyst contains 27.1 wt % less iridium than benchmark IrO_2_, and around 7 times higher Ir-mass activity.^66^ Note that the activity of 6H-SrIrO_3_ has been measured by several groups, reporting different values; see Table S1. For instance, mass-normalized activities of 49, 75, and 40 A g^–1^_Ir_ have been reported at 1.55, 1.525, and 1.5 V, respectively; see Table S1. Most likely, the origin of the scattered activity values stems in the different protocols used for the assessment of the OER activity in RDE, mostly the mass of Ir deposited on the electrode, but also the electrolyte, activation protocol, etc. These features will be discussed below.

9R-BaIrO_3_ has an Ir-mass activity of 168 A g^–1^_Ir_ at 1.5 V, which is around 16 times higher than that of IrO_2_ (10 A g^–1^_Ir_), and it only requires 1.46 V to reach 10 mA cm^–2^_geo_.^67^ In order to explain the higher activity of 9R-BaIrO_3_ we turn our attention to recent studies in the literature suggesting that oxides with small Ir–Ir distances display high OER activity.^33,63^ This hypothesis would explain why 9R-BaIrO_3_, with Ir–Ir distances of 2.616 Å, is more active than 6H-SrIrO_3_ with Ir–Ir distances of 2.7696 Å. However, more studies involving oxides with either shorter or lager Ir–Ir distances are needed to confirm the soundness of this hypothesis. For instance, it would be worth studying oxides such as Ba_4_Ir_3_O_10_ and Ba_7_Ir_6_O_19_, which display small Ir–Ir distances of 2.591 and 2.557 Å, respectively.

Doping 6H-SrIrO_3_ with Co, Ni, Fe, or Cu has been reported to increase the OER activity of 6H-SrIrO_3_.^68^ The origin of such improvement is not clear, and it may account for several factors. For instance, the higher OER activity of Co-doped oxide is related to its higher surface area of the doped oxide, due to the lower synthesis temperature, compared to the undoped material. In addition, Co leads to an increase in the coverage of surface hydroxyl groups, which regulates the Ir–O bond covalency, and modulates the oxygen *p*-band center, reaching 1.465 V at 10 mA cm^–2^_geo_.^68^ The partial substitution of Ir by Mn in 9R-BaIr_1–*x*_Mn_*x*_O_3_ leads to an activity enhancement of 73 times compared to IrO_2_.^69^ Finally, a triple hexagonal perovskite Ba_3_TiIr_2_O_9_ (A_3_M′M″_2_O_9_), with similar structure than 6H-SrIrO_3_, records an OER activity of 1.505 V at 10 mA cm^–2^_geo_.^70^

### Group 2 Iridates

**Figure 4:**
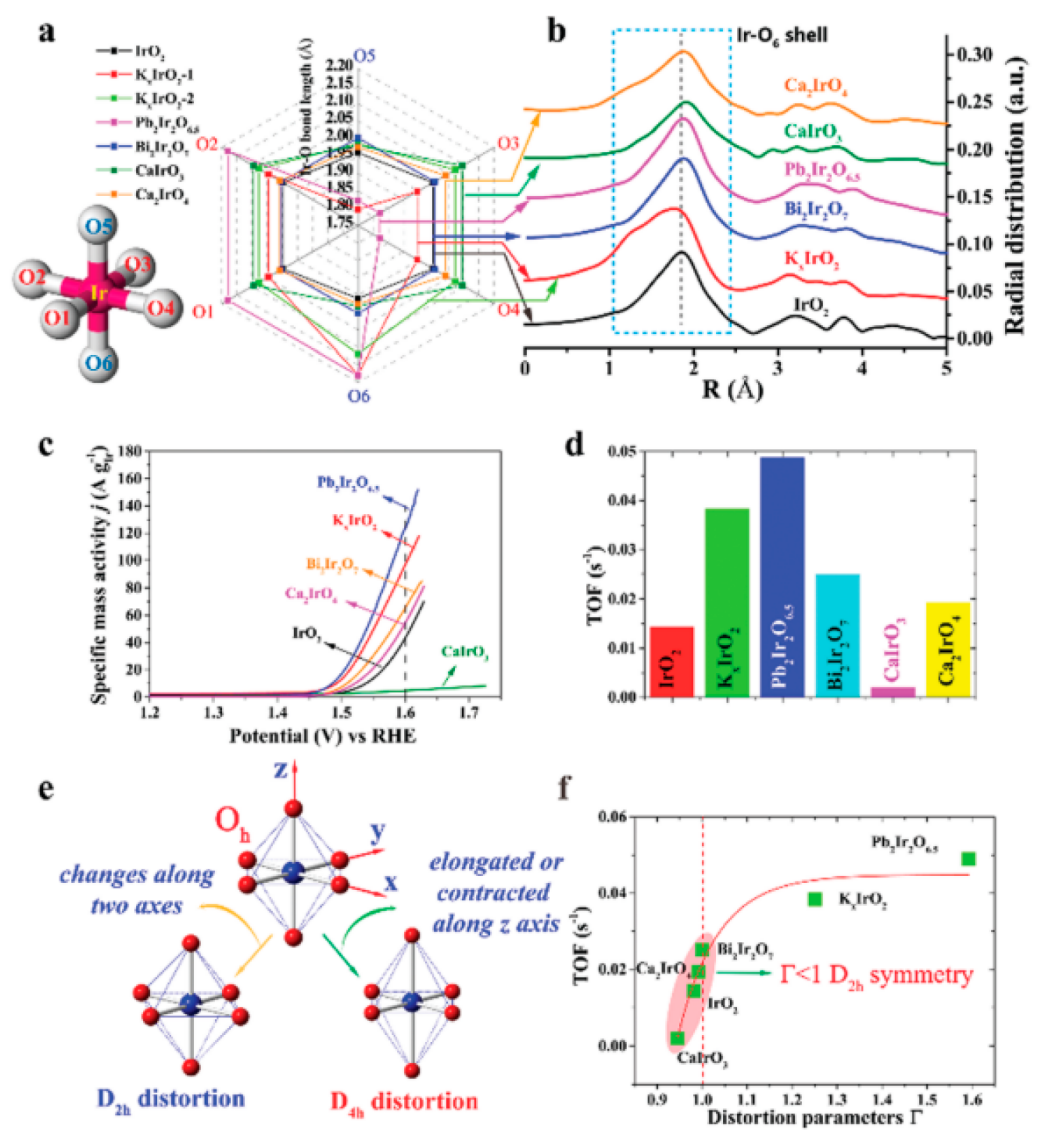
(a) Ir–O bond length distributions of IrO_6_ on different oxides and (b) their corresponding *k*^0^-normalized Ir-L_III_ edge EXAFS values. (c) *i*R corrected specific mass activity of the oxides. The scan rate is 10 mV s^–1^, the mass loading of all catalysts is 0.2 mg cm^–2^, and the electrode area is 0.25 cm^–2^. (d) A comparison of TOF values of the different catalysts at η = 0.37 V. (e) Different IrO_6_ distortion types. (f) Relationship between the TOF and the distortion of the Oh in terms of a distortion parameter (Γ). Larger Γ values mean larger distortion. Reproduced with permission from ref (34). Copyright 2019 Royal Society of Chemistry.

This group includes iridates in which IrO_6_ octahedra are connected through the edges. The oxides in this group can be arranged into:

(i) Compounds with IrO_6_ edge-shared in columns, such as Ca_2_IrO_4_ and Ca_5_Ir_3_O_12_. Ca_2_IrO_4_ crystallizes in a hexagonal symmetry with IrO_6_ significantly distorted. Ca_2_IrO_4_ and Ca_2_Y_*x*_Ir_1–x_O_4_ have been reported to display high OER activity, achieving 1.443 V at 10 mA cm^–2^_geo_ or 632.6 A g^–1^_Ir_ at 1.5 V for Ca_2_Y_0.2_Ir_0.8_O_4_, which is among the best values reported in literature for any Ir compound.^71^ The authors proposed that the OER activity is strongly affected by the distortion of the IrO_6_ octahedra. This is an interesting observation, which also applies to Ir-mixed oxides from other groups. For instance, we observed that the hexagonal perovskites in group 1, which usually display high OER activities, also present highly distorted IrO_6_ octahedra. Sun et al.^72^ studied the OER activity of several iridates, namely, Pb_2_Ir_2_O_7_, K_*x*_IrO_2_, Bi_2_Ir_2_O_7_, Ca_2_IrO_4_, IrO_2_, and CaIrO_3_, and observed that the OER activity increases with the distortion of the Oh (Figure 4). They claim that higher IrO_6_ distortion (particularly in a D_4h_ elongation), makes electrons near the Fermi level more delocalized. The electrons near *E*_F_ are very important for surface-oxygen bonding, so their delocalization reduces the adsorption energy of the determining step (formation of OOH* intermediates), being beneficial to the overall conductivity of the catalyst and to the OER activity.

To the best of our knowledge, the activity of Ca_5_Ir_3_O_12_ has not been measured hitherto. Since its structure is very similar to that of Ca_2_IrO_4_, with both materials showing similar Ir–Ir distances (3.195 and 3.194 Å) and very similar IrO_6_ distortions, we would expect both catalysts to display similar OER activity.

(ii) Layered compounds that include A_2_IrO_3_ (A = Na, Li, Cu) with several polymorphs, MgIrO_3_, Na_0.5_IrO_3_, etc. In general, these compounds consist of layers of edge-sharing IrO_6_ and A ions situated in the interlayer spacing. There are several iridates that share layers and corners, such as KIrO_3_ with a KSbO_3_ structure, CaIrO_3_ with a pyroxene structure, KIr_4_O_8_ with a hollandite structure, etc. To the best of our knowledge, none of these materials has been studied as OER catalysts.

### Group 3 Iridates

This group is the largest and most studied group for the use of OER catalysts. It includes iridates with IrO_6_ connected through the corners. Iridates in this group can adopt three main kinds of structures, perovskites, pyrochlores, and Ruddlesden–Popper oxides.

3C-SrIrO_3_ has a perovskite structure and can be regarded as the parent compound of this group of iridates. The OER activity of 3C-SrIrO_3_ and of its doped counterparts, SrIr_1–*x*_M_*x*_O_3_, M = Zn, Zr, Co, Ti, Sc, and Cr, has been measured by several groups.^73−78^ 6H-SrIrO_3_ (which belongs to group 1 and has an Ir–Ir distance of 2.7696 Å), is more active than 3C-SrIrO_3_ (Ir–Ir 3.896 Å), probably due to the shorter Ir–Ir bonds inside the face-sharing dimers in 6H-SrIrO_3_.^66^ It is possible to enhance the OER activity of 3C-SrIrO_3_ by doping it with transition metal cations in the Ir site without modifying the connectivity between the IrO_6_ units. SrIr_1–*x*_Zr_*x*_O_3_ reaches a potential of 1.47 V at 10 mA cm^–2^_geo_ (1540 A g^–1^_Ir_ at 1.525 V). This high OER activity is attributed to the reduction on the particle size compared to SrIrO_3_, and the weakening of surface oxygen-binding.^74^ SrTi_1–*x*_Ir_*x*_O_3_, which contains 57 wt % less iridium than IrO_2_ reaches 1.477 V at 10 mA cm^–2^_geo_ (820 A g^–1^_Ir_ at 1.525 V). The authors claim that Ir dopants in SrTiO_3_ activate the intrinsically inert titanium sites into sites even more active than Ir.^75^ SrIr_1–*x*_Cr_*x*_O_3_ records a very low potential of 1.447 V at 10 cm^–2^_geo_ (417.6 A g^–1^_Ir_ at 1.525 V), which is attributed to an amorphous layer of CrIrO_*x*_ with edge-shared CrO_*x*_ and IrO_*x*_ octahedra formed upon the fast leaching of Sr during the OER.^78^

**Figure 5:**
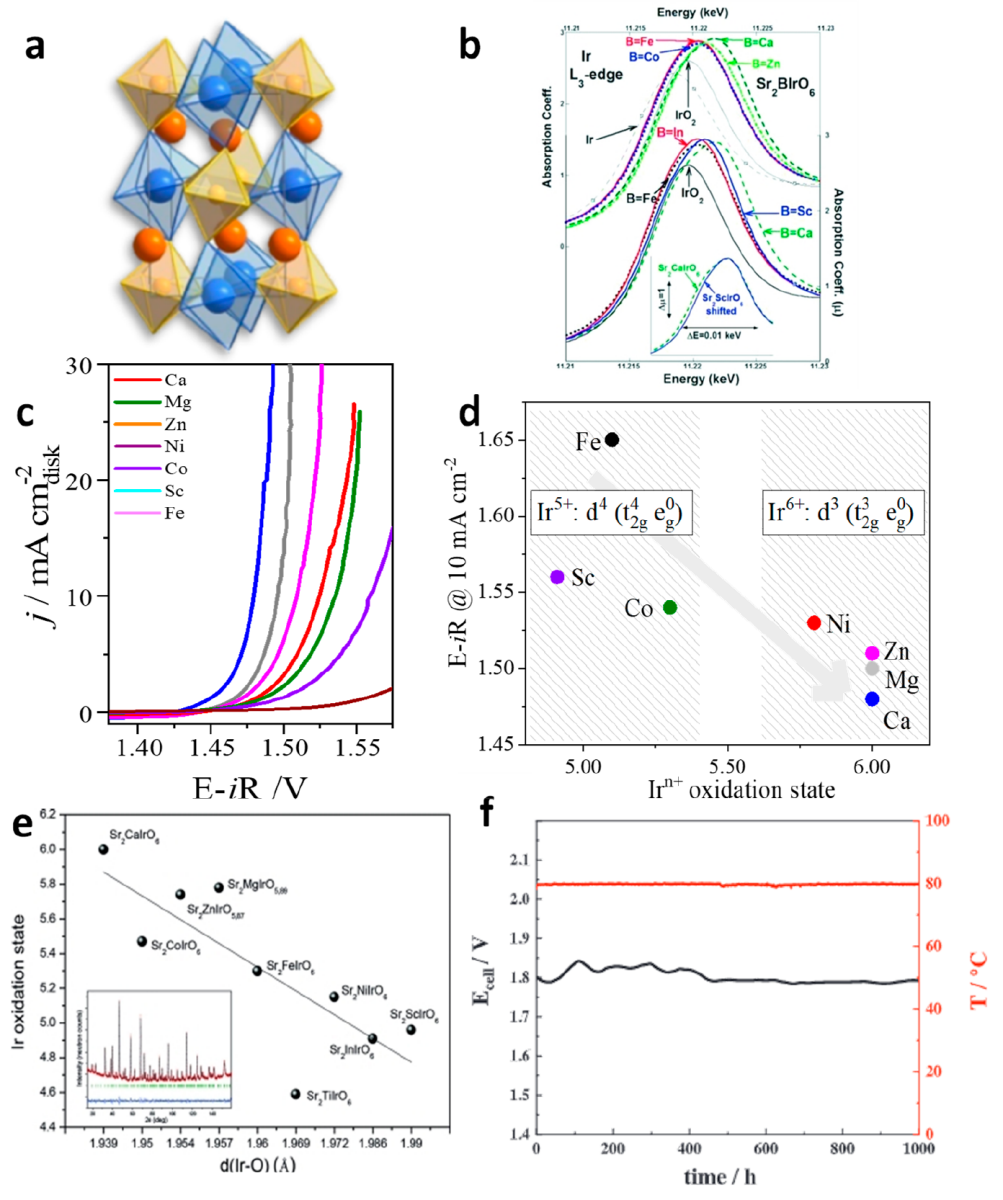
(a) Crystal structure of a distorted double perovskite in which IrO_6_ and MO_6_ octahedra share corners. Reproduced with permission from ref (80). Copyright 2022 Springer Nature. (b) Ir L_3_-edge XAS spectra Sr_2_BIrO_6_ with B = Ca, Fe, Co, Zn (top) and Sc, Ca, Ni and Zn (bottom) along with those of elemental Ir and IrO_2_. Reproduced with permission from ref (83). Copyright 2015 Wiley-VCH Verlag GmbH & Co. (c) Current densities of Sr_2_MIrO_6_ (M = Ca, Mg, Zn, Ni, Co, Sc and Fe) measured at 1600 rpm in O_2_-saturated 0.1 M HClO_4_ using catalyst loading of 0.25 mg_cat_ cm^–2^ and a scan rate of 10 mV s^–1^. Adapted with permission from ref (81), Copyright 2021 Royal Society of Chemistry and ref (80), Copyright 2022 Springer Nature. (d) Influence of the Ir^n+^ oxidation state of each double perovskites on the OER potentials necessary to reach 10 mA cm^–2^. Adapted with permission from ref (81). Copyright 2021 Royal Society of Chemistry. Oxidation state and activity data for Zn, Mg, and Ca adapted with permission from ref (80). Copyright 2022 Springer Nature. (e) Correlation between the distortion of the IrO_6_ octahedra and the Ir^n+^ oxidation state of different iridates. Reproduced with permission from ref (83). Copyright 2015 Wiley-VCH Verlag GmbH & Co.. (f) Evolution of the cell potential (*E*_cell_) and cell temperature (*T*_cell_) during a PEMWE measurement (1000 h) at constant 2 A cm^–2^ using Sr_2_CaIrO_6_ and Pt/C as anode and cathode respectively. Reproduced with permission from ref (84). Copyright 2023 Wiley-VCH GmbH.

Double perovskites with the general formula A_2_MIrO_6_ (A= Ca, Sr and Ba; M = transition metals), in which Ir and M cations are situated in two different crystallographic sites (Figure 5a), have been studied; see, for instance, La_2_LiIrO_6_, Ba_2_RIrO_6_ (R = Y, La, Ce, Pr, Nd, Tb) and Sr_2_MIrO_6_ (M= Ni, Fe, Co, Sc, Mg, Zn, Ca).^19,79−81^ In the case of La_2_LiIrO_6_, the authors demonstrated that the Ir^5+^–O bond is not active for the OER. The formation of an oxidized Ir surface at pH 1 leads to an enhanced OER activity.^19^ The effect of the oxidation state of Ir cations in mixed oxides is a topic of discussion in the literature. Rutile IrO_2_, with Ir^4+^ cations in IrO_6_ Oh, is the benchmark catalyst for the OER in acidic media. However, theoretical studies reveal that α-IrO_3_, with Ir^6+^ cations in corner-sharing octahedra, have higher OER activity (thermodynamic) than rutile IrO_2_.^82^ In addition, Pourbaix diagrams of the Ir–H_2_O phase reveal that α-IrO_3_ is also stable under acid conditions. However, the stabilization of Ir^6+^ cations in IrO_3_ oxides is not possible without the involvement of other cations, for instance, via intercalation of Li^+^ or through the formation of Ir-mixed oxides. Due to the high oxidation state of Ir^6+^ cations, these compounds are formed only under high oxygen pressures of 200 bar. More importantly, by properly choosing the nature of the nonmetal (M) cation(s), it is possible to synthesize Ir-mixed oxides with Ir oxidation states ranging between 4+ and 6+. In our group, we have studied the OER activity of a series of double perovskites with the general formula Sr_2_MIrO_6_ with M = Fe, Co, Ni, Sc, Zn, Mg, Ca.^80,81^ As shown in Figure 5c, the OER activity in the RDE varies with M, with Sr_2_CaIrO_6_ and Sr_2_FeIrO_6_ recording the highest and the lowest OER activities, respectively. The OER activity increases with the increasing oxidation state of Ir, with Ir^6+^ (Sr_2_CaIrO_6_) showing the highest activity of 900 A g^–1^_Ir_ at 1.525 V (Figure 5d).^80^ This trend is supported by theoretical studies.^81^ Ir’s oxidation state is related with the Ir–O distances^83^ (see Figure 5e), which are shortened with the high oxidation states, and with the hybridization between Ir and O orbitals. In addition, and in good agreement with results discussed above, the OER activity of Sr_2_MIrO_6_ increases with the distortion of the IrO_6_ octahedra.^80^ When measured in PEMWE configuration, Sr_2_CaIrO_6_ delivers very high activity, 2 A g_Ir_^–1^ at 1.8 V during at least 1000 h (see Figure 5f).^84^

In order to assess the effect of Ir’s oxidation state for the OER, further structures should be studied, such as Ba_2_MIrO_6_ and Ca_2_MIrO_6_, with M being a cation in the 2+ oxidation state.

Oxides other than perovskites can also display a network of corner-sharing IrO_6_ octahedra. Pyrochlores with the general formula A_2_B_2_O_7_ are one such oxide. Pyrochlores crystallize in the cubic, face-centered space group *Fd-*3*m*, where the smaller B cations are 6-fold coordinated in trigonal antiprism distorted IrO_6_ octahedra with all the six oxygen anions at equal distance from the central cation. There are many iridates with a pyrochlore structure and with A = Bi, Pb, Bi, Eu, Nd, Pr, Y, Yb, Tl cations and B = Ir and Ru, Mn, Y cations, etc.; many of them have been studied as electrocatalysts for the OER in acidic media. In the pyrochlores family, the first conclusion that can be made is that the introduction of Ru in the Ir sublattice improves the activity in terms of potential, with Y_2_Ru_1.2_Ir_0.8_O_7_ reaching 1.45 V at 10 mA cm^–2^.^85^ This improvement can be rationalized by keeping in mind that Ru active sites are more active than Ir active sites, although they are less stable.

**Figure 6:**
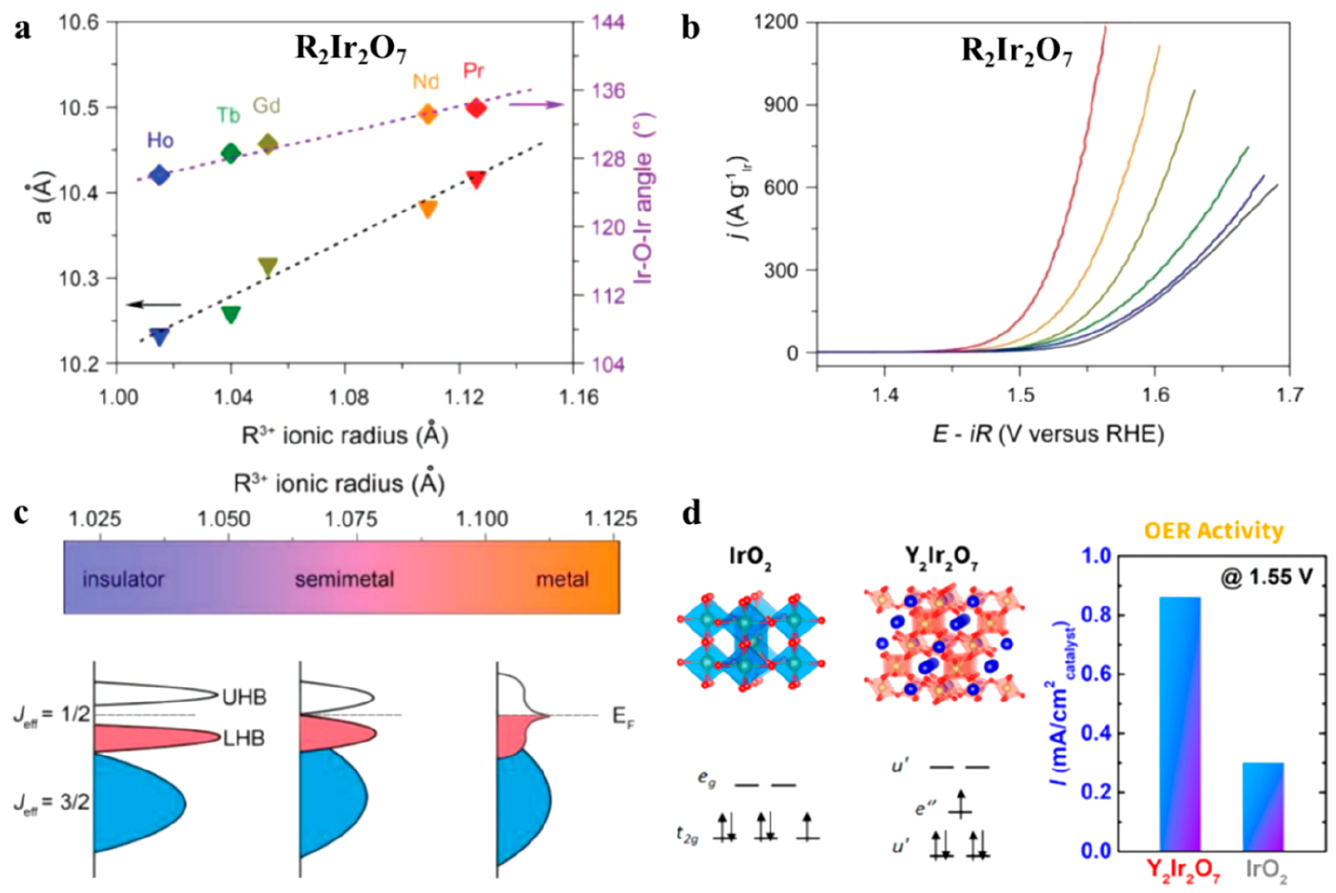
(a) Effect of the size of the A-site cation on the Ir–O–Ir bond angles and lattice parameter *a* of several pyrochlore iridates (R_2_Ir_2_O_7_, R= Ho, Tb, Gd, Nd, and Pr). (b) OER mass activities recorded with the different iridates in 0.1 M HClO_4_. (c) Electronic phase diagram around room temperature and the corresponding schematic band structures of Ir 5*d* orbitals. Figures a, b, and c reproduced with permission from ref (86). Copyright 2018 Wiley-VCH Verlag GmbH & Co. (d) Electron filling in the e″ orbital of Ir 5*d* states and its contribution to the improved OER activity of Y_2_Ir_2_O_7_. Reproduced from ref (87). Copyright 2018 American Chemical Society.

The effect of the cation A on the activity of pyrochlores was evaluated in R_2_Ir_2_O_7_ (R = Ho^3+^, Tb^3+^, Gd^3+^, Nd^3+^, and Pr^3+^).^86^ The OER activity increased from 36.2 to 424.5 A g_Ir_^–1^ at 1.525 V by increasing the ionic radius of R and the Ir–O–Ir angle, i.e., for Ho_2_Ir_2_O_7_ to Pr_2_Ir_2_O_7_, respectively; see Figure 6a and b. The higher kinetics were ascribed to the insulator to metal transition and the enhancement of the Ir–O bond covalency as the R size increases (Figure 6c).

Other features have also been reported to influence the OER activity of pyrochlores. For instance, the high activity of Y_2_Ir_2_O_7_ (41.1 A g_Ir_^–1^ at 1.525 V) can be related to the electron filling on the last band of the transition metal cations, as shown in Figure 6d. This proposal relies on previous works which relate the OER activity of mixed oxides in alkaline media with the e_g_ electron occupancy (higher activity for e_g_ = 1) of the active transition metals cations on various perovskites.^88^ In the above pyrochlore, it has been reported that the J_eff_ splitting of the t_2g_ due to the spin orbit coupling (SOC) induces a single-electron filling in the e″ (J_eff_) orbital of the Ir 5*d* states, which could contribute to the improved OER activity.^87^ The high OER activity at an e_g_ occupancy close to unity was predicted to be related with a high covalency of the transition metal–oxygen bonds.^89^

Ruddlesden–Popper iridates display a general formula of A_*m*+1_Ir_*m*_O_3*m*+1_ (*m* = 1, 2, 3, etc.) in a layered structure. The corner-shared IrO_6_ are only in two-dimensions for *m* = 1 or two consecutive layers depending on *m*, in an arrangement similar to perovskites inside the layers and the SrO structure in between them. This structure leads to weaker connectivity than perovskites.

Sr_3_Ir_2_O_7_ has been reported as the most active electrocatalyst among the Ruddlesden–Popper iridates, displaying a mass-normalized activity of 160 A g_Ir_^–1^ at 1.525 V. Its activity has been correlated with its high conductivity and an optimized binding energy.^90^ Also, Sr_2_IrO_4_ has being reported very active by some authors achieving 394 A g_Ir_^–1^ at 1.55 V.^44^ Its high activity has been ascribed to the stable Ir surface formed due to Sr leaching. Note, however, that other authors reported lower Ir mass-normalized activities for the same oxide, with values of 70 A g_Ir_^–1^ at 1.525 V,^90^ yet displaying similar current density and potential at 10 mA cm^–2^_geo_ (see Table S1). Again, the origin of the very different mass-normalized activities can be found in the experimental protocol used to test the OER activity.

The high activity of layered Ruddlesden–Popper iridates has been also attributed to the easily protonation of the material due to its layered conformation.^91^

Finally, R_3_MO_7_ is another family of materials with a structure derived from the fluorite structure. These compounds are formed by a one-dimensional column of corner-sharing Ir octahedra, crystallizing in an orthorhombic symmetry. Sm_3_IrO_7_ has been tested as OER catalyst, offering 307 A g_Ir_^–1^ at 1.525 V.^92^ In their work, the authors claim that the active species for the OER are IrO_*x*_/Sm_3_IrO_7_ rather than Sm_3_IrO_7_.

### Group 4 Iridates

Ir-mixed oxides in this group display a network of nonconnected (isolated) IrO_6_ octahedra. The number of mixed oxides in group 4 is small, including R_3_IrO_7_ (R= La, Nd, Pr, etc.); A_4_IrO_6_ (A = Ca, Sr) with a K_4_CdCl_6_ structure, and also Li_8_IrO_6_. To our knowledge, only Sr_4_IrO_6_, displaying a hexagonal cell with IrO_6_ octahedra separated by Sr atoms, has been tested for OER, reaching 274 A g_Ir_^–1^ at 1.55 V.^44^ In that work, the activity of Sr_4_IrO_6_ was lower than the activities reported for iridates displaying connectivity between their IrO_6_ units.

Although less common, mixed oxide structures in which the Ir cations do not occupy octahedral positions have also been reported. For instance, in the spinel structure (AM_2_O_4_) the cations occupy octahedra (MO_6_) and tetrahedra (AO_4_) positions. There are very few examples of spinels with 4*d* and 5*d* cations in A or M sites.^93^ For Ir, only LiIr_2_O_4_ and ZnIr_2_O_4_ have been reported to display the spinel structure, but they have been only synthesized as epitaxially grown thin films.^94,95^ Among spinels, to the best of our knowledge, only Ir-doped NiCo_2_O_4_ with IrO_4_ in tetrahedra has been reported active for the OER in acidic media.^96^ There are also few oxides reporting Ir in square-planar IrO_4_ coordination, such as Na_4_IrO_4_ and K_4_IrO_4_ compounds, that can be worth to be studied.^97^

In summary, Ir-mixed oxides with very high Ir mass-normalized activities in the RDE can be obtained. In order to understand the origin of their high activity, we have analyzed their OER activity based on features such as the connectivity of the IrO_6_ octahedra, which in turs affects the Ir–Ir and Ir–O distances, oxidation state of the Ir, IrO_6_ distortions, conductivity, density of states, hybridization, SOC, Ir–O–Ir angles, etc.

## Dissolution and Reconstruction during OER

In order to be suitable for practical applications, Ir-mixed oxides should combine high activity and durability. Whereas obtaining electrocatalysts with high OER activity is possible, see above, increasing the stability of Ir-mixed oxides is a still a grand challenge, since even pure IrO_2_ dissolves at potentials above 1.6 V.^98^ Moreover, the dissolution processes may be exacerbated by the presence of non-noble metal elements in Ir-mixed oxides. As a result, almost all, if not all, Ir-mixed oxides are unstable during the OER operation and tend to dissolve and reconstruct during the process.

**Figure 7:**
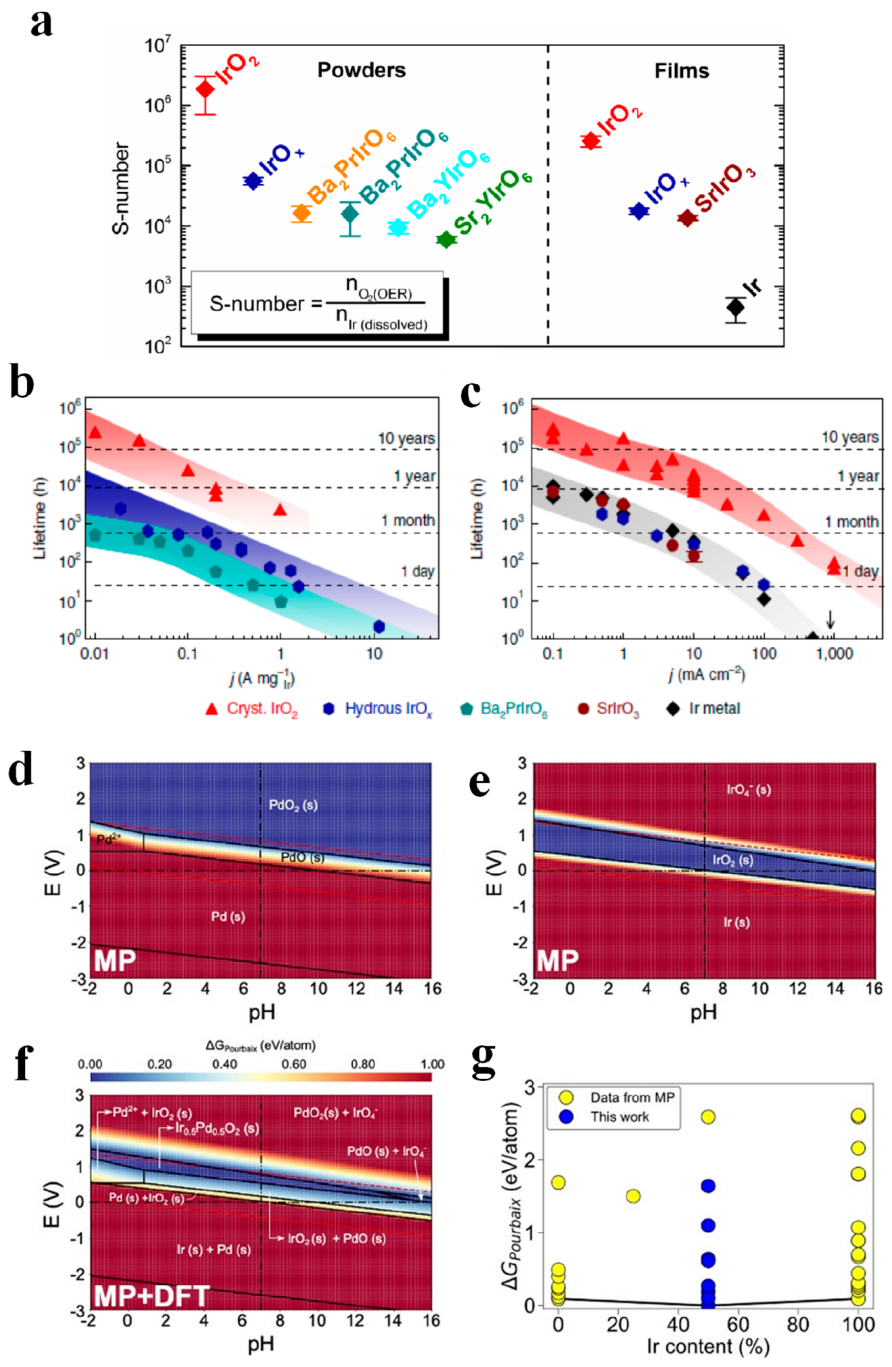
(a) Stability-number calculated for different catalysts as powders and films in 0.1 M HClO_4_ (slow scan rate of 5 mV s^–1^ to 1.55 V vs RHE and 1.65 V vs RHE for films and powders respectively). (b, c) Lifetime estimated for the different powders (b) and films (c) based on the use of the stability number. Figures a, b, and c reproduced with permission from ref (99). Copyright 2018 Springer Nature. (d, e) Pourbaix diagrams for monometallic Pd and Ir with respect to the water system (the color indicates the value of Δ*G*_pbx_ for one of the phases with respect to the other phases; bluer color means closer values of Δ*G*_pbx_ to zero). (f) Pourbaix diagram for bimetallic Ir–Pd oxides. (g) Convex hull diagram of Δ*G*_Pourbaix_ at pH = 0 and *E* = 1.23 V_RHE_. Yellow and blue symbols are data taken from Materials Project and calculated in ref (104). Figures d, e, f, and g reproduced from ref (104). Copyright 2020 American Chemical Society.

The quantification of the dissolution of Ir, or the non-noble elements, has been observed and quantified in several papers, mostly by analyzing the content of the element dissolved in the electrolyte and its evolution with time. Recently it has been proposed that it is possible to predict the durability of electrocatalysts during the OER from the ratio between evolved oxygen and the amount of dissolved iridium. This metric, which has been proposed by several groups, is referred to as S-number^99^ or activity stability factor.^100^ As shown in Figure 7a–c, the highest S–numbers were obtained for crystalline IrO_2_, with Ir-mixed oxides displaying 2 orders of magnitude lower S-number, hence lower durability. However, since the evolution of metal dissolution was conducted during a somehow short period of time, this metric fails to consider the possible stabilization of the phases formed during operation, a feature that has actually been reported for Ir double perovskites.^80,84^ Also, it is important to remark that S-numbers obtained in aqueous electrolyte with Ir-catalysts are significantly smaller (by ca. 5 orders of magnitude) than the ones obtained in PEMWE configuration.^101^

In order to achieve a rational design of advanced catalysts, it would be desirable to be able to predict the stability of Ir-mixed oxides under typical OER conditions. In principle, this is possible by constructing Pourbaix diagrams, which allow us to predict the thermodynamic stable phases of a chemical species based on pH and E. However, constructing Pourbaix diagrams based upon experimental results for all possible mixed oxides is not feasible. To address this issue (and others), the Massachusetts Institute of Technology and Lawrence Berkeley National Laboratory collaborated to create the Materials Project. This initiative has resulted in the development of a vast database, which enables the construction of Pourbaix diagrams using computational data for thousands of compounds , including those that have not yet been synthesized.^102^ Recently, a new approach that quantitatively evaluates the thermodynamic stability of a phase by comparing the difference in Gibbs free energies (Δ*G*_pbx_) such as phase vs potential and pH with respect to the stable phase has been proposed. A stable material should display Δ*G*_pbx_ = 0; however, it is suggested that Δ*G*_pbx_ below 0.5 eV atom^–1^ would suffice to suggest that the material would be stable under the studied conditions.^103^Figure 7 demonstrates how DFT, Δ*G*_pbx_, and the Materials Project database can be used to predict and discover the stability of new acid-stable compounds. The Pourbaix diagrams for monometallic Pd and Ir with respect to the water system are shown in Figure 7d and e. Figure 7f displays the Pourbaix diagram for bimetallic Ir–Pd oxides, with the color indicating the value of Δ*G*_pbx_ for one of the phases with respect to the other phases. The bluer the color is, the closer Δ*G*_pbx_ is to zero, indicating greater stability. From Figure 7d–f, it is possible to predict that when Ir and Pd are combined to form Ir_0.5_Pd_0.5_O_2_, the resulting compound is even more stable than the monometallic phases, especially in regions close to 2 V.

This approach improves the accuracy of predicting thermodynamic stability, especially for catalysts that are not theoretically in a region of high stability. However, it is important to note that kinetic factors are not considered when Pourbaix diagrams are used to study stability. Just because a material is stable in a particular phase or has a low Δ*G*_pbx_ does not guarantee that it will be kinetically active for the OER. However, the complexity of mixed oxides makes it challenging to identify which phases these materials can form, and interpreting diagrams with many phases can be difficult. Therefore, while Pourbaix diagrams are useful, their limitations should be considered, especially when dealing with mixed oxides. Another way to achieve a rational design of stable and active catalysts for the OER in acid is through the use of machine learning. However, it requires a precise predictive model, accurate input information, and high-quality data obtained via DFT or experimental methods. Furthermore, a large amount of data is necessary for training. To ensure accurate predictions, it is essential to validate the results experimentally and continue training the system with the obtained values.

Regardless of the actual structure and composition, metal-based mixed oxides are prone to dissolution in an acidic electrolyte. Several strategies are being considered to prevent and delay the dissolution of mixed oxides. Recent studies suggest that is possible to increases Ru’s stability during OER by doping with monovalent elements in A sites of the perovskite.^105,106^ To our knowledge, this approach has not been proved with Ir-mixed oxides.

The dissolution of the non-noble metal and Ir from mixed oxides has profound implications for the OER activity. On the one hand, since Ir is lost during the OER, the durability of the catalysts becomes compromised. On the other hand, the dissolution leads to the reconstruction of the mixed oxide,^99,105,107^ so the actual structure and composition of the reconstructed solid, especially at the surface level, may be completely different to that of the as synthesized material. Although in general after reconstruction, a more or less amorphous surface layer of Ir-oxides is formed, there is no universal reconstruction pathway. The extension of the reconstruction and the nature of the reconstructed material depend on the structure, composition, and morphology of the as prepared Ir-mixed oxide.

Among the Ir-mixed oxides reported in the literature, hexagonal perovskites with strong IrO_6_ connectivity between columns exhibit the highest level of OER stability. According to Yang et al.,^66^ the presence of face-sharing octahedra in 6H-SrIrO_3_ prevents Sr dissolution, and only ∼1% of total Sr content is leached after 30 h of OER, whereas ∼24% of total Sr content is leached from 3C-SrIrO_3_. In this line, it has been reported that Co-doped 6H-SrIrO_3_ is stable during the OER, without reporting surface reconstruction.^68^ The high stability of 9R-BaIrO_3_ has also been attributed to the IrO_6_ connections, with 9R-BaIrO_3_ being more stable than 3C-SrIrO_3_, and even more stable than iridates with lower connectivity.^69^

Other works, however, report the formation of IrO_*x*_ nanoparticles at the surface of 9R-BaIrO_3_ during the OER cycles. After further OER cycling, the surface evolves into amorphous Ir^4+^O_*x*_H_*y*_/IrO_6_ octahedra and then to amorphous Ir^5+^O_*x*_/IrO_6_ octahedra on the surface.^67^ Mn-doping in 9R-BaIrO_3_ promotes the dissolution of Ir, so there is a good balance between Ba- and Ir-dissolution, and a robust and thin surface layer of BaIr_1–*x*_Mn_*x*_O_3_ with strong IrO_6_ connectivity is formed at the surface.^69^

**Figure 8:**
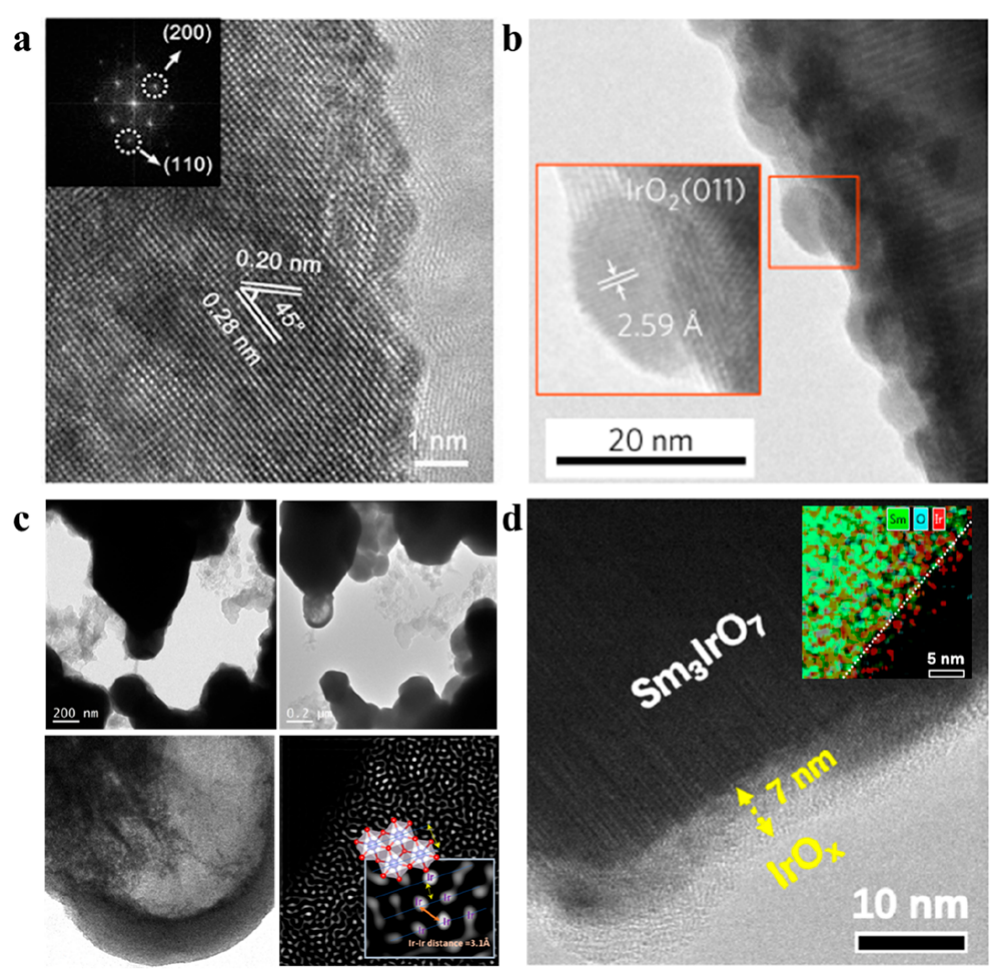
Representative TEM micrographs of Ir-mixed oxides after the OER cycles. (a) SrTi_1–*x*_Ir_*x*_O_3_ without surface restructuration after OER. Reproduced with permission from ref (75). Copyright 2019 Wiley-VCH Verlag GmbH & Co. KGaA. (b) Example of the formation of crystalline IrO_2_ nanoparticles La_2_LiIrO_6_. Reproduced with permission from ref (19). Copyright 2016 Springer Nature Limited. (c) IL-TEM images showing the evolution of the same regions during OER. HRTEM and aberration corrected TEM images showing hollow particles of dimers of edge-shared IrO_6_ octahedra generated upon the reconstruction of Sr_2_CaIrO_6_ after 5000 OER cycles. Reproduced with permission from ref (80). Copyright 2022 Springer Nature. (d) Amorphous layer of IrO_*x*_ formed on Sm_3_IrO_7_. Reproduced with permission from ref (92). Copyright 2023 American Chemical Society.

Concerning the perovskite catalysts; SrTi_1–*x*_Ir_*x*_O_3_ is the only catalyst reported to display highly crystallinity after OER, without surface amorphourization (Figure 8a).^75^ The formation of surface layers of amorphous IrO_*x*_ during OER has been reported for 3C-SrIrO_3_ and 3C-BaIrO_3_,^108,109^ similar to SrCo_0.9_Ir_0.1_O_3-δ_, with range-ordered amorphous IrO_*x*_ layers,^73^ and Sr_2_CoIrO_6_, Sr_2_FeIrO_6_ and Sr_2_Fe_0.5_Ir_0.5_O_4_.^81,110^ SrIr_0.8_Zn_0.2_O_3_ perovskite also forms an Ir-rich amorphous phase, that after OER cycles becomes a resistive material, which is no longer electrochemically accessible.^76^ In the case of Ba-based double perovskites, the leaching of the non-noble elements leads to the formation of highly active amorphous iridium oxide.^99^ La_2_LiIrO_6_ evolves into IrO_2_ particles at the surface;^19^ these particles are small but crystalline (see Figure 8b).

The actual restructuring experienced by iridium-mixed oxides during the OER depends not only on their structure but also on their composition. This is clearly observed by following the reconstruction of a family of double perovskites with the same structure, Sr_2_MIrO_6_, in which only the cation M is modified. Whereas Sr_2_FeIrO_6_ particles display the characteristic crystallographic features of the initial perovskite after 50 OER cycles, the composition, morphology, and structure of Sr_2_NiIrO_6_ is strongly affected, and after 50 OER cycles the dissolution of Ni and Sr leads to the collapse of the perovskite particles and the formation of ill-crystalline nanosized IrO_*x*_ particles.^81^ Due to the very fast dissolution of Ca and Sr, Sr_2_CaIrO_6_ experiences a different restructuring pattern, which commences already upon immersion in aqueous acidic electrolyte (0.1 M HClO_4_). The rapid dissolution of the cations results in the formation of hollow particles of corner- and edge-sharing IrO_6_ dimers, in a very open structure;^80^ see Figure 8c. However, the morphology of the particles is not strongly affected, as observed with IL-TEM, and the formation of nanoparticles of IrO_*x*_H_*y*_ is not observed. Once formed, this open structure remains very active and stable during OER, as observed from the long-term experiments conducted in RDE and PEMWE configurations; see Figure 5f and discussion above.^84^ The restructuration observed on Sr_2_CaIrO_6_ is somehow similar to the previously observed for SrIr_1–*x*_Cr_*x*_O_3_.^78^ In both cases, the very fast leaching of Ca and Sr cations leads to surfaces rich in edge-shared Ir-oxygen octahedra. In this kind of reconstructions, leading to the formation of hollow surfaces, it has been also reported that the voids formed during the reconstructions are rapidly filled by hydroxonium (H_3_O^+^) ions,^91,111−113^ thereby stabilizing the hollow structure of nanosized (short-range order) clusters of IrO_6_ octahedra.

Concerning the pyrochlores, Hubert et al.^114^ reported that cation dissolution during electrochemical testing, resulted in the formation of a surface layer of IrO_*x*_ on Y_2_Ir_2_O_7_. The Ir oxidation state at the surface is dynamic, with Ir more oxidized than the bulk. In ref (115), the formation of a highly active IrO_*x*_ surface layer due to leaching of the Y^3+^ cations into the electrolyte solution was also reported. In Lu_2_Ir_2_O_7_, a surface reconstruction into a metastable [IrO_6_]–[IrO_6_] framework occurs due to Lu^3+^ leaching. The reconstructed IrO_*x*_/Lu_2_Ir_2_O_7_ structure contributes to the broadening of the t_2g_ bandwidth, which reflects the downshift of the *d*-band center. The delocalized feature of Ir 5*d* conducted the reduced *p*–*d* separation and the reduced energy gap, which demonstrated the enhanced Ir–O covalency (enhance the Ir–O hybridization) and the enhanced conductivity of the [IrO_6_]–[IrO_6_] framework. After several OER cycles, such an active framework evolves into an amorphous layer with low conductivity, that is the main cause for the decreased of the OER activity.^116^ In Sm_3_IrO_7_, also IrO_*x*_ is formed at the surface (Figure 8d).^92^

Ruddlesden–Popper Sr_2_IrO_4_ reconstructs in corner-shared and under-coordinated IrO_6_ octahedra, responsible for their high activities.^91^ Finally, in oxides such as Ca_4_IrO_6_ and Sr_4_IrO_6_, in which the IrO_6_ octahedra are completely isolated with no connection between them, a rapid disintegration of the crystal lattice can be anticipated if Ca and Sr leach out in an acid solution.^63^

To summarize, during the OER, Ir-mixed oxides undergo a reconstruction process triggered by the dissolution of the cations in their structure. As a result, the composition and structure of the mixed oxide are modified in one (or several) of the following ways. The vast majority of mixed oxides develop a surface layer of amorphous IrO_*x*_, that in most cases continuously evolves until the materials deactivates, probably due to the complete dissolution of IrO_*x*_ or due to the formation of a resistive and inert surface. In other oxides, e.g., Sr_2_FeIrO_6_ and La_2_LiIrO_6_, the dissolution of the non-noble cations leads to the formation of IrO_2_ nanoparticles, either deposited on the surface of the oxide or as isolated nanoparticles. A third group of materials in which the non-noble metal cations leach very fast, typically Ca, suffers a very fast restructuration into hollow and very active structures with edge-shared IrO_6_ octahedra. Finally, a small group of oxides, mainly face-shared hexagonal perovskites such as SrTi_1–*x*_Ir_*x*_O_3_ and Y_2_Ir_2_O_7_, appear to be stable during the OER.

These reconstructions are not compartmentalized, and in many cases, they are interrelated processes taking place more or less simultaneously during the different stages of the OER. In fact, since the actual nature of the outermost layers of the reconstructed catalysts is difficult to be determined accurately, further advanced characterization studies are needed to understand the reconstruction of Ir-mixed oxides. In this sense, in situ/operando techniques, typically XRD, XAS, and XPS, but also IL-TEM, can offer invaluable information about the reconstruction pattern of mixed oxides during the OER, while allowing to identify the nature of the surface (and bulk) species responsible for the OER activity.^33,98,117−121^

## Recommended Protocols for Measuring OER Activity in Mixed Oxides

In the previous sections, we have identified a number of discrepancies between activity data reported in the literature for identical catalysts from different laboratories. This is a major hurdle for the proper identification and understanding of the parameters that control the OER activity of mixed oxides and hence for the rational design of advanced OER electrocatalysts. In part, these discrepancies account for the wide range of protocols used for the assessment of the OER activity. RDE is an effective and cost-efficient method for prescreening novel electrocatalysts for the OER. As shown in this perspective, almost all of the studies published about Ir-mixed oxides for the OER report data obtained only from RDE.

Standardized protocols for measuring catalyst activity for OER in RDE in acidic electrolyte have been reported,^122^ but mixed oxides may undergo significant restructuring during the measurement, altering their crystallographic structure. Some researchers report electrochemical activities and restructuring in acidic media before or after applying a potential program, making it difficult to compare catalysts across studies and identify the active phase and true activity for the OER. As such, protocols must be expanded and adapted for mixed oxides.

**Figure 9:**
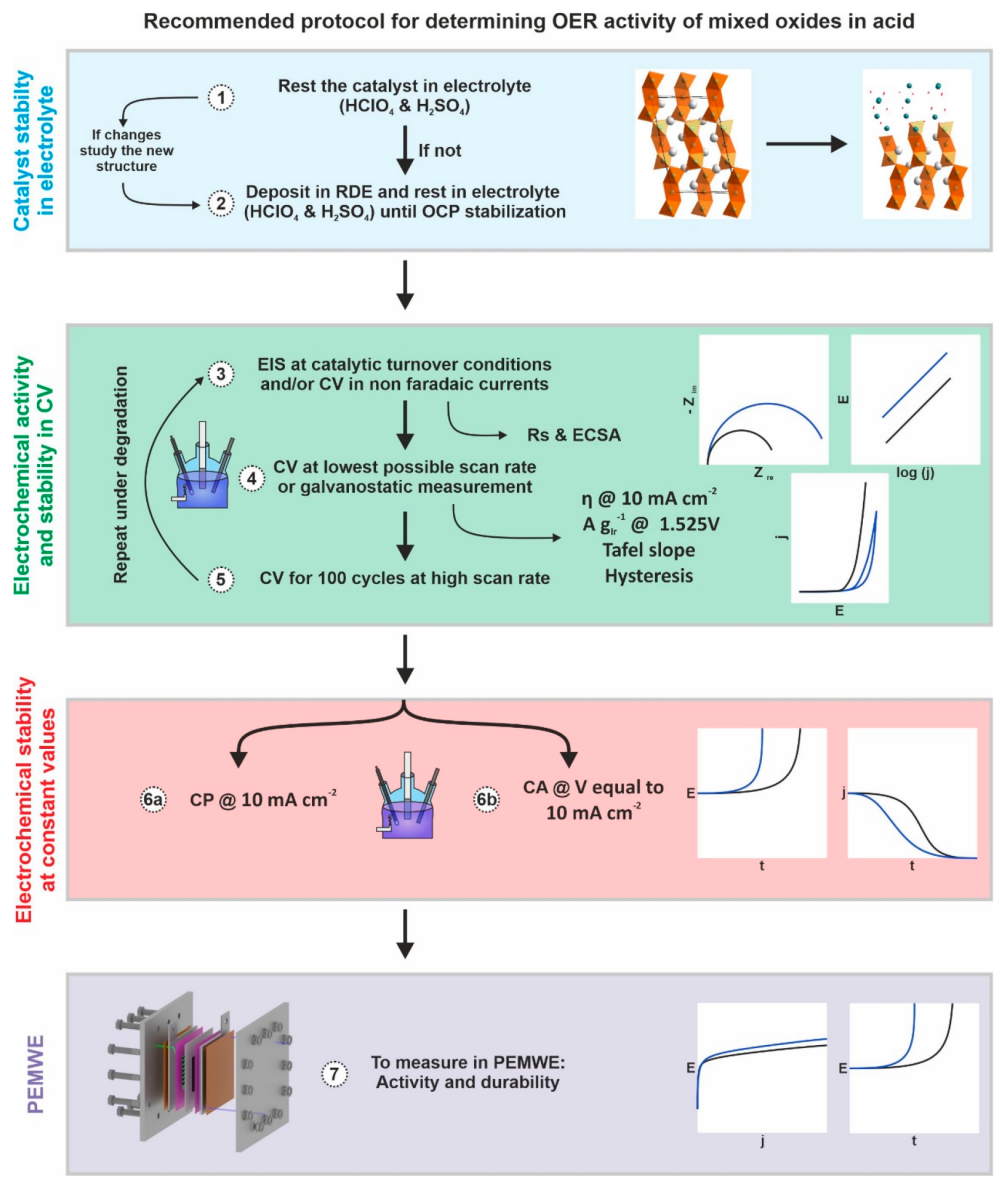
Proposed protocol for the assessment of the OER activity of mixed oxides in acidic electrolyte, including steps for evaluating electrocatalyst stability in acidic electrolyte (step 1), RDE activity and durability (steps 3–6), and activity in PEMWE (step 7).

We propose a protocol outlined in Figure 9 for studying mixed oxide catalysts. Step 1 involves immersing the synthesized catalyst powder in acidic electrolyte for at least 60 min, washing, and characterizing it to determine the stability of the crystal structure. This crucial step is rarely performed. Note for instance, that non-noble cations can dissolve already during the preparation of the ink. The choice of acid for RDE measurements is a topic of debate,^123^ with some studies suggesting that HClO_4_ may be better than H_2_SO_4_ due to the strong adsorption of SO_4_^2–^ ions on the surface of the catalyst.^124^ The counterion effect may be more pronounced in mixed oxides, where the use of H_2_SO_4_ can lead to precipitation with leached cations and passivation.^125^ Therefore, it is recommended to investigate the effect of each acid on the new material.

After assessing the catalyst stability in the electrolyte and determining its nature, step 2 is to deposit the catalyst on the RDE (0.255 mg_cat_ cm^–2^), immerse the electrode in an O_2_-saturated electrolyte, and wait for the open circuit potential (OCP) to stabilize. The OCP stability indicates the stability of the electrode–electrolyte interface. The next steps follow a similar protocol proposed previously.^122^ Step 3 is to measure electrochemical impedance spectroscopy (EIS) at a potential where electrocatalysis takes place. At these values, it is possible to determine *R*_s_ and use it to account for the ohmic resistance (*i*R) during the measurement of the OER activity. We have noticed that throughout the articles cited in this work, different % of *i*R compensations have been used without any justification. Unless properly justified, 100% of the *i*R should be used to correct activity curves.^126^ Additionally, by EIS we can calculate the double layer capacitance (*C*_dl_), which will allow us to determine the electrochemical surface area (ECSA) to later determine the intrinsic activity of the catalysts under study. While it is possible to estimate the ECSA using voltammetric measurements, it can be challenging to do so for mixed oxides. In these cases, it is recommended to use EIS instead of voltammetric measurements.^127,128^ EIS allows the capacitive component to be considered as a Constant Phase Element (CPE). This approach not only allows for the determination of *C*_dl_ but also accounts for the nature of the surface roughness.^129^ Furthermore, if required, EIS facilitates conducting more comprehensive studies,^130^ enabling the investigation of the physicochemical properties of the material at the nanoscale, similar to those examined in supercapacitors.^131,132^ Moreover, measuring the CPE by EIS allows us to study the surface reconstructions and to determine the evolution of the surface area during the OER cycles (see an example in SI Figure S3).

Once the EIS measurement has been carried out, the next step (step 4) is to study the catalytic activity with cyclic voltammetry (CV) at a low scan rate of 10 mV s^–1^. This technique allows to extract the potential at 10 mA cm^–2^ and the Ir mass-normalized activity, i.e., the OER activity per gram of Ir on the electrode (expressed in A g^–1^_Ir_) at a potential of 1.525 V. These values are important for comparing different catalysts.^133^ Generally, this last value is given at a potential of 1.525 V, although in some cases, other values are reported. While this value varies strongly every few mV, we recommend always extracting the value at 1.525 V for better comparability among different mixed oxides. Across the papers, obtaining accurate and precise data can be challenging, and it is recommended that numerical values be reported rather than just graphical data. Precise data can be analyzed using machine learning techniques to predict new active catalysts, and this is an area of growing interest.

It is important to collect cyclic voltammograms (CV) instead of applying a linear sweep voltammogram (LSV) program since the former may reveal the hysteresis due to secondary reactions or reconstruction of the surface. Sometimes it is recommended to use steady-state chronoamperometry to measure activity at different overpotentials, as the influence of oxygen intercalation may overestimate the activity.^31^ Furthermore, performing steady-state measurements allows minimization of the contribution of the capacitive current. Correcting nonfaradaic current should be avoided when side reactions are present, as this correction is only to eliminate the *C*_dl_. With the CV, it is possible to extract the Tafel slope to understand, a priori, the reaction mechanism. Additionally, it is advisible to extract the slope in at least two decades,^134^ except in cases where there is a transition between one rate determination step and another.^135^

Once the initial characterization is completed using EIS (step 3) and CV (step 4) at a low scan rate or at steady-state chronoamperometry, step 5 involves conducting stability measurements of the catalyst using CV on the same electrode and examining its degradation during the cycles. For this purpose, a given number of CV cycles should be carried out at a faster scan rate, such as 100 cycles at 100 mV s^–1^. Following that, steps 3 and 4 are repeated. Conducting step 3 after step 5 allows one to assess the variations of *R*_ct_ and *C*_dl_, which can indicate structural modifications throughout the cycles. The repetition of step 4 after certain cycles permits evaluation of the evolution of catalytic activity with the OER cycles and identification of possible changes in Tafel slopes. This whole sequence (steps 3–5) should be repeated until several thousands of OER cycles are recorded or until the OER activity declines.

Finally, in step 6, for a better assessment of the durability of a catalyst, after depositing the catalyst again onto the RDE, it is preferable to continue studying its activity degradation using chronopotentiometry at a current of 10 mA cm^–2^ or chronoamperometry at an equivalent potential to the initial CV of 10 mA cm^–2^.

## From RDE to PEM

It is worth noting that conducting measurements using an RDE provides a quick and relatively effective method for screening a material’s activity and durability. However, measurements taken using an RDE have certain limitations. First, the currents that can be measured are much lower than those in PEMWE. Second, catalysts are much more durable in PEMWE than in RDE due to various factors such as the accumulation of bubbles on the surface of the RDE.^136^ Although similar formations occur in MEA, it is to a lesser extent.^137^ As mentioned before, another factor to consider is the effect of counterions, which may affect the catalyst to a varying extent. Lastly, the effect of pH or high potentials on the glassy carbon or the carbon where the catalyst is dispersed can create a passivating/corrode layer, further affecting the measurement.^138^ Therefore, while measurements taken using RDE are effective for determining a catalyst’s activity, it is highly recommended to measure catalysts using PEMWE for a more accurate representation of their activity and durability in real industrial operating conditions^101,139^ or in a gas diffusion electrode (GDE) setup as presented recently in reference.^140^

In summary, in this Perspective, we have shown that Ir-mixed oxides with a wide range of compositions and structures can be synthesized. Regardless of their initial composition and structure, the most active mixed oxides display a potential of around, or slightly lower than, 1.5 V at 10 mA cm^–2^, in RDE. This value is similar to that reported for state-of-the-art IrO_*x*_ catalysts. When normalized to the Ir content, however, a number of Ir-mixed oxides display higher mass-normalized OER activities than IrO_2_ (see Table S1 and Figure S2), making them promising candidates to replace IrO_*x*_ as electrocatalysts in PEMWEs. Even if an unequivocal conclusion cannot be reached and more iridates need to be studied, the results above distillate a number of structural features relevant for the OER activity of Ir-mixed oxides. Thus, iridates with high OER activity tend to display short Ir–Ir distances, high Ir oxidation state, distorted IrO_6_ octahedra and a strengthened hybridization between Ir 5*d* and O 2*p* orbitals that enhances conductivity. However, establishing proper structure–activity descriptors from the as synthesized Ir-mixed oxides may be misleading. In order to identify the true nature of the active phases, it should be considered that Ir-mixed oxides will undergo a reconstruction process during the EOR triggered by leaching of the non-noble elements. It is therefore imperative to study and understand this reconstruction and identify the nature of the species (surface and bulk) in the reconstructed phase. It is also desirable to unify a protocol for measuring and reporting the OER activity of novel catalysts, including RDE and PEMWE measurements. This will allow construction of accurate and accessible libraries with accurate activity data that can be used to identify accurate structure–activity descriptors that will allow for a rational design of Ir-based catalyst for the OER.

## Supporting Information Available

Tables and figures collating the OER activities of Ir-based mixed oxides in RDE and PEMEW; evolution of the double-layer capacitance during OER cycling (PDF)
