# Supplementary material for: Unraveling the Most Relevant Features for the Design of Iridium Mixed Oxides with High Activity and Durability for the Oxygen Evolution Reaction in Acidic Media
Source: JACS Au. 2023 Aug 23;3(9):2336–55. doi: 10.1021/jacsau.3c00247 (PMC10523372; doi:10.1021/jacsau.3c00247)
Supplement: Supplementary file 1 — au3c00247_si_001.pdf [file au3c00247_si_001.pdf]

Unraveling the Most Relevant Features for the Design of Iridium Mixed Oxides with High Activity and Durability for the Oxygen Evolution Reaction in Acidic Media

Dmitry Galyamin,[⁋] Álvaro Tolosana-Moranchel, [⁋] María Retuerto*, Sergio Rojas*

Grupo de Energía y Química Sostenibles. Instituto de Catálisis y Petroleoquímica. CSIC. C/ Marie Curie 2. 28049 Madrid, Spain

Table S1. OER activities reported for Ir-based mixed oxides of group 1 (hexagonal perovskites), group 2, group 3 (perovskites, pyrochlores, Ruddlesden-Popper) group 4 and with other crystal structures in RDE.

Group 1

Hexagonal perovskites

Catalyst	**Ir oxidation state**	**Mixed oxide Loading (mg cm^{-2})**	**Ir wt.%**	**Ir loading in RDE (mg cm^{-2})**	**E-iR / V @ 10 mAcm^{-2}**	**Tafel slope mV dec^{-1}**	**i_M A g^{-1}_{Ir} @ 1.525 V**	**BET / m^2 g^{-1}**	**Durability tests**	**Electrolyte**	**Reconstruction studied**	**Reference**
**6H-$SrIrO_3$**	Ir^{4+}	0.08	59	0.0472	1.583	63-91	49 @ 1.55	3.2	6 h @ 10 mA cm^{-2}	0.1 M $HClO_4$	Yes	ACS Appl. Energy Mater. 2019, 2, 8, 5490–5498
**6H-$SrIrO_3$**		0.9	59	0.531	1.478		75	0.3	30 h @ 10 mAcm^{-2}	0.5 M H2SO4	Yes	Nat Commun 9, 5236 (2018)
**6H-$SrIrO_3$**		0.45			1.482	58.7	75			0.1 M $HClO_4$	No	ACS Appl. Mater. Interfaces 2019, 11, 45, 42006–42013
**Co-doped 6H-$SrIrO_3$**					1.465	51.8	170				No	
**Fe-doped 6H-$SrIrO_3$**					1.52		20					
**Ni-doped 6H-$SrIrO_3$**					1.485		80					
**Cu-doped 6H-$SrIrO_3$**					1.46		100					
**Zn-doped 6H-$SrIrO_3$**					1.5		90					
**6H-$SrIrO_3$**		0.5	59	0.295	1.575	58	10 @ 1.60	1.42	12h @ 10 Ag^{-1}	0.5 M H_2SO_4	No	Chem 5, 3243–3259, 12, 2019
**9R-$BaIrO_3$**			57	0.285	1.525	51.5	10 @ 1.58	1.32	24 h @ 10 Ag^{-1}			
**$Ba_3TiIr_2O_9$**		0.281	39	0.10959	1.505	45.7	250 @1.53 V		20 h @ 10 mAcm^{-2}	0.1 M $HClO_4$	Yes	Chem. Mater. 2020, 32, 3904−3910
**6H-$SrIrO_3$**		0.277			1.53	122	40 @ 1.5		5 h @ 10 mA cm^{-2}	0,5 M H_2SO_4	Yes	J. Am. Chem. Soc. 2021, 143 (43), 18001–18009.
**IrO_x/9R-$BaIrO_3$**					1.46	80	168 @ 1.5		48 h @ 10 mA cm^{-2}			

Grupo 2

Catalyst	Ir oxidation state	Mixed oxide Loading (mg cm^{-2})	Ir wt.%	Ir loading in RDE (mg cm^{-2})	E-iR / V @ 10 mAcm^{-2}	Tafel slope mV dec^{-1}	i_M A g^{-1}_{Ir} @ 1.525 V	BET / m^2 g^{-1}	Durability tests	Electrolyte	Reconstruction studied	Reference
**Ca_2IrO_4**	Ir^{4+}, Ir^{5+}	0.2	57	0.114	1.56		57.02		6 h @ 1.6V	0.1 M $HClO_4$	No	ACS Omega 2018, 3, 2902−2908
**Ca_2IrO_4**		0.04	57	0.0228			11			0.1 M $HClO_4$	No	Chem. Commun., 2019, 55, 5801-5804
**Ca_2IrO_4**		0.283	57	0.161	1.48	56.6	135,5 @ 1.5		168 h @ 10 mA cm^{-2}	1 M $HClO_4$	No	ACS Energy Lett. 2022, 7, 11, 3798–3806
**$Ca_2Y_{0.1}Ir_{0.9}O_4$**			57	0.161	1.49	60	100 @ 1.5					
**$Ca_2Y_{0.2}Ir_{0.8}O_4$**			55	0.156	1.443	44.5	632,6 @ 1.5					
**$Ca_2Y_{0.3}Ir_{0.7}O_4$**			51	0.144	1.505	82	75 @ 1.5					

<u>Group 3</u>

Perovskites

Catalyst	Ir oxidation state	Mixed oxide Loading (mg cm^{-2})	Ir wt.%	Ir loading in RDE (mg cm^{-2})	E-iR / V @ 10 mAcm^{-2}	Tafel slope mV dec^{-1}	i_M A g^{-1}_{Ir} @ 1.525 V	BET / m^2 g^{-1}	Durability tests	Electrolyte	Reconstruction studied	Reference
**IrOx/$SrIrO_3$**	Ir^{4+}		59						30 h @ 10 mAcm^{-2}	0.5 M H_2SO_4	Yes	Science 2016, 353, 1011-1014
**La_2LiIrO_6**	Ir^{4+}, Ir^{5+}	0.255	34	0.0867	1.53	50	33.5	1.7	50 cycles bt. 1.1-1.7 V @ 10 mVs^{-1}	H_2SO_4	Yes	Nat Energy 2, 16189 (2017)
**Ba_2YIrO_6**	Ir^{4+}, Ir^{5+}	0.015	14	0.0021		67-196			1h @ 10 mAcm^{-2}	0.1 M $HClO_4$	Yes	Nat Commun 7, 12363 (2016)
**Ba_2LaIrO_6**			20	0.003		59-127						
**Ba_2CeIrO_6**			20	0.003		57-121						
**Ba_2PrIrO_6**			20	0.003	1.63	54-106						
**Ba_2NdIrO_6**			20	0.003	1.61	59-136						
**Ba_2TbIrO_6**			22	0.0033		61-118						

**3C-$SrIrO_3$**		0.9	59	0.531	1.5		35	0.3	30 h @ 10 $mAcm^{-2}$	0.5 M H2SO4	Yes	Nat Commun 9, 5236 (2018)
**$SrCo_{0.9}Ir_{0.1}O_{3-\delta}$**	Ir^{4+}	0.255	9	0.0230	1.55		67	0.1	3 h @ 10 $mAcm^{-2}$	0.1 M $HClO_4$	Yes	Nature Commnications (2019) 10:572
**$SrTi_{0.67}Ir_{0.33}O_3$**	Ir^{4+}	0.21	27	0.0567	1.477	45-80	820	45	20 h @ 10 $mAcm^{-2}$	0.1 M $HClO_4$	Yes	Angew.Chem. Int.Ed. 2019, 58,7631 –7635
**$CaIrO_3$**		0.04	60	0.024						0.1 M $HClO_4$	No	Chem. Commun., 2019, 55, 5801-5804
**$SrZrO_3$:$SrIrO_3$ (Zr:Ir 1:2)**		0.13	1.47			43	1540		1000 cycles	0.1 M $HClO_4$	No	Adv.Mater.2020, 32, 2001430
**Sr_2NiIrO_6**	Ir^{6+}, Ir^{5+}	0.255	37	0.09435	1.525	48	115		1 h @ 10 $mAcm^{-2}$	0.1 M $HClO_4$	Yes	J. Mater. Chem. A, 2021, 9, 2980–2990
**Sr_2CoIrO_6**			37	0.09384	1.535	52	73	1.2	10000 cycles bt. 1.2-1.7 V @ 50 mVs^{-1}			
**Sr_2ScIrO_6**			38	0.09639	1.56	67	35		1 h @ 10 $mAcm^{-2}$			
**Sr_2FeIrO_6**			37	0.09435	1.65	90	5.6	0.3	1 h @ 10 $mAcm^{-2}$			
**$SrIr_{0.8}Zn_{0.2}O_3$**		0.08	54	0.0432	1.57		110 @ 1,55	4	900 cycles bt. 1.065-1.865 V @ 20 mVs^{-1}	0.1 M $HClO_4$	Yes	J. Am. Chem. Soc. 2021, 143, 9961−9971
**$SrCo_{0.5}Ir_{0.5}O_3$**	Ir^{4+}, Ir^{5+}	0.255	37	0.09435	1.51	41.5	84.76 @ 1.5 V	0.18	50 cycles bt. 1.2-1.6 V	0.1 M $HClO_4$	Yes	Sci. Adv. 7, eabk1788 (2021)
**$SrSc_{0.5}Ir_{0.5}O_3$**			38	0.0969	1.53	40.4	36.30 @ 1.5 V	0.53				
**Cr-$SrIrO_3$**	Ir4+	0.34	57	0.1938	1.447	54	417.6		40 h @ 10 $mAcm^{-2}$	0.1 M $HClO_4$	Yes	Nano Energy 102 (2022) 107680
**IrO_2/$LiLa_2IrO_6$**	Ir^{4+}, Ir^{5+}	0.416			1.508	45			13 h @ 10 $mAcm^{-2}$	0.1 M $HClO_4$	Yes	J. Mater. Chem. A, 2022, 10, 3393–3399
**Sr_2CaIrO_6**	Ir^{6+}, Ir^{5+}	0.25	38	0.095	1.485	33	900		5000 CV cycles	0.1 M $HClO_4$	Yes	Nature Commun., 2022, 13:7935
**Sr_2MgIrO_6**			39	0.0975	1.505	33	260					
**Sr_2ZnIrO_6**			36	0.09	1.518	38	139					
**$SrIrO_3$**		0.283	59	0.16697	1.539	61	20	1.54		0.5 H_2SO_4	No	Chem. Eng. J. 423 (2021) 130185

Pyrochlores

Catalyst	Ir oxidation state	Mixed oxide Loading (mg cm^{-2})	Ir wt.%	Ir loading in RDE (mg cm^{-2})	E-iR / V @ 10 mAcm^{-2}	Tafel slope mV dec^{-1}	i_M A g^{-1}_{Ir} @ 1.525 V	BET / m^2 g^{-1}	Durability tests	Electrolyte	Reconstruction studied	Reference
**$Bi_2Ir_2O_7$**	Ir^{4+}, Ir^{5+}	0.402	42	0.16884	1.58	45	1.19 @ 1.5 V		1000 cycles bt. 1.1-1.6 V @ 50 mVs^{-1}	1 M H_2SO_4	Yes	Chem. Mater. 2012, 24, 4192−4200
**$Bi_2Ir_2O_7$**	Ir^{4+}	0.102	42	0.04284		45	26	30	500 cycles bt. 1.0-1.6 V @ 50 mVs^{-1}	0.1 M $HClO_4$	No	Chem. Mater. 2017, 29, 5182−5191
**$Y_2Ir_2O_7$**			57	0.05814		50	34	21				
**$Pb_2Ir_2O_7$**			42	0.04284		65	9	24				
**$BiYIr_2O_7$**			48	0.04896		40	18	28				
**$BiPbIr_2O_7$**			42	0.04284		42	13	38				
**$YPbIr_2O_7$**			49	0.04998		41	13	11				
**$Y_2Ir_2O_7$**		0.16	57	0.0912	1.575	51.8	41.1	7.3	24h @ 10 mA cm^{-2}	0.1 M $HClO_4$	Yes	ACS Appl. Energy Mater. 2018, 1, 8, 3992–3998
**$Pr_2Ir_2O_7$**	Ir^{4+}	0.057	49	0.02793	1.52		424.5	1.55	1000 cycles bt. 1-1.55 V @ 50 mVs^{-1}	0.1 M $HClO_4$	Yes	Adv.Mater.2019, 31, 1805104
**$Nd_2Ir_2O_7$**			49	0.02793	1.55		156.3	1.53	1000 cycles bt. 1-1.55 V @ 50 mVs-1 and 10000s @ 10 mA cm^{-2}			
**$Gd_2Ir_2O_7$**			47	0.02679	1.58		73	2.58				
**$Tb_2Ir_2O_7$**			47	0.02679	1.62		51.2	3.2				
**$Ho_2Ir_2O_7$**			47	0.02679	1.64		36.2	4.7				
**$Bi_2Ir_2O_7$**		0.04	42	0.0168			18			0.1 M $HClO_4$	No	Chem. Commun., 2019, 55, 5801-5804
**$Pb_2Ir_2O_{6.5}$**			43	0.0172			36					
**$Yb_2Ru_{1.4}Ir_{0.6}O_7$**	Ir^{4+}	0.1	13	0.013	1.51	47				0.1 M $HClO_4$	No	Electrochimica Acta 366 (2021) 137327
**$Yb_2Ru_{1.16}Ir_{0.84}O_7$**			19	0.019	1.48	50						
**$Yb_2Ru_{0.90}Ir_{1.10}O_7$**			25	0.025	1.486	55						
**$Yb_2Ru_{0.30}Ir_{1.7}O_7$**			39	0.039	1.555	59						
**$Gd_2Ru_{1.46}Ir_{0.54}O_7$**			13	0.013	1.52	55						

**$Gd_2Ru_{1.1}Ir_{0.9}O_7$**			21	0.021	1.5	58						
**$Gd_2Ru_{0.90}Ir_{1.10}O_7$**			26	0.026	1.508	50						
**$Gd_2Ru_{0.34}Ir_{1.66}O_7$**			39	0.039	1.544	44						
**$Nd_2Ru_{1.46}Ir_{0.54}O_7$**			13	0.013	1.492	50						
**$Nd_2Ru_{1.16}Ir_{0.84}O_7$**			20	0.02	1.504	51						
**$Nd_2Ru_{0.84}Ir_{1.16}O_7$**			28	0.028	1.508	55						
**$Nd_2Ru_{0.32}Ir_{1.68}O_7$**			41	0.041	1.558	--						
**$Lu_2Ir_2O_7$**		0.2	45	0.09	1.535	42.8	220 @ 1.58 V	7.8	5,55 h @ 10 mA cm^{-2}	0.1 M $HClO_4$		ACS Appl. Mater. Interfaces 2021, 13 (25), 29654–29663.
**$Y_2Ir_2O_7$**	Ir^{4+}, Ir^{5+}	0.015	57	0.00855	1.584		249 @ 1.55 V	22	2 h @ 1.6 V_{RHE}	0.1 M $HClO_4$		J. Phys. Chem. C 2022, 126, 1751−1760
**$Y_2Ir_2O_7$**	Ir^{4+}, Ir^{5+}				1.647		38 @ 1.55 V			0.5 M H_2SO_4	Yes	
**$Y_2Ru_{1.2}Ir_{0.8}O_7$**	Ir^{3+}, Ir^{4+}	0.51	22	0.1122	1.45	47.56			2000 h @ 10 $mAcm^{-2}$	0.5 M H_2SO_4	Yes	Small 2022, 18, 2202513
**Ruddlesden-Popper**												
**Sr_2IrO_4**			45	0.036	1.516	45-73	394 @ 1.55	2.8	6 h @ 10 mA cm^{-2}	0.1 M $HClO_4$	Yes	ACS Appl. Energy Mater. 2019, 2, 8, 5490–5498
**Sr_2IrO_4 --> $H_{3.6}IrO_4$**		0.255	46	0.1173					1 h @ 1 mA cm^{-2}_{oxide}		No	Chem. Mater. 2020, 32, 3499−3509
**Sr_2IrO_4**			45	0.12735	1.514	57	70	1.40		0.5 H_2SO_4	No	Chem. Eng. J. 423 (2021) 130185
**$Sr_3Ir_2O_7$**			51	0.14433	1.489	50	160	0.78	1000 CV cycles			
**$Sr_2Ru_{0.8}Ir_{0.2}O_4$**	Ir^{4+}	0.0255	10	0.00255	1.58	55	1100 @ 1.6 V_{RHE}		24 h @10 mA cm^{-2}	0.1 M $HClO_4$	No	J. Energy Chem. 70 (2022) 623–629
**$Sr_2Ru_{0.6}Ir_{0.4}O_4$**			22	0.00561	1.57	49	1400 @ 1.6 V_{RHE}					
**$Sr_2Ru_{0.5}Ir_{0.5}O_4$**			28	0.00714	1.566	47	1450 @ 1.6 V_{RHE}					
**$Sr_2Ru_{0.4}Ir_{0.6}O_4$**			35	0.008925	1.575	51	1400 @ 1.6 V_{RHE}					
**$Sr_2Ru_{0.2}Ir_{0.8}O_4$**			50	0.01275	1.58	58	1350 @ 1.6 V_{RHE}					
**Sr_2IrO_4**			67	0.017085		59	700 @ 1.6 V_{RHE}					

Group 4

Catalyst	Ir oxidation state	Mixed oxide Loading (mg cm^{-2})	Ir wt.%	Ir loading in RDE (mg cm^{-2})	E-iR / V @ 10 $mAcm^{-2}$	Tafel slope mV dec^{-1}	i_M A g^{-1}_{Ir} @ 1.525 V	BET / m^2 g^{-1}	Durability tests	Electrolyte	Reconstruction studied	Reference
**Sr_4IrO_6**	Ir^{4+}	0.08	30	0.024	1.517	50-134	274 @ 1.55	12	6 h @ 10 mA cm^{-2}	0.1 M $HClO_4$	Yes	ACS Appl. Energy Mater. 2019, 2, 8, 5490–5498

Other crystal structures

Catalyst	Ir oxidation state	Mixed oxide Loading (mg cm^{-2})	Ir wt.%	Ir loading in RDE (mg cm^{-2})	E-iR / V @ 10 $mAcm^{-2}$	Tafel slope mV dec^{-1}	i_M A g^{-1}_{Ir} @ 1.525 V	BET / m^2 g^{-1}	Durability tests	Electrolyte	Reconstruction studied	Reference
**$BaIrO_{2.937}/La_3IrO_7$**	Ir^{4+}, Ir^{5+}	0.416			1.52	66.2	561.6 @ 1.63 V_{RHE}		7 h @ 45 mA cm^{-2}	0.1 M $HClO_4$	Yes	Inorg. Chem. Front., 2022,9, 702-708
**Sm_3IrO_7**		0.57	25	0.1425	1.532	48	307		10 h @ 10 mA cm^{-2}	0.1 M $HClO_4$	No	ACS Appl. Mater. Interfaces 2023, 15, 11, 14282–14290
**$Ir-NiCoO_4$**		0.2	26	0.052	1.51	40	380 @ 1.55 V_{RHE}	67.9	5.5 h @ 10 mA cm^{-2}	0.1 M $HClO_4$	No	Appl. Catal. B: Environ. 244 (2019) 295–302
**K_xIrO_2**		0.04					30			0.1 M $HClO_4$	No	Chem. Commun. 2019, 55, 5801-5804

Ir oxide doped catalysts

Catalyst	Ir oxidation state	Mixed oxide Loading (mg cm^{-2})	Ir wt.%	Ir loading in RDE (mg cm^{-2})	E-iR / V @ 10 $mAcm^{-2}$	Tafel slope mV dec^{-1}	i_M A g^{-1}_{Ir} @ 1.525 V	BET / m^2 g^{-1}	Durability tests	Electrolyte	Reconstruction studied	Reference
**$Li–IrO_x$**		0.05	--	--	1.53	39	100	27	10 h @ 10 mA cm^{-2}	0.5 H_2SO_4	No	J. Am. Chem. Soc. 2019, 141, 7, 3014–3023
**$IrHf_xO_y$**					1.56	60	6950 A g_{Ox}^{-1}		6 h @ 5 mA cm^{-2}	0.1 M $HClO_4$	Yes	J. Am. Chem. Soc. 2021, 143, 38, 15616–15623
**Ta0.1Tm0.1Ir0.8Ox**		0.041			1.428	64		93.6	500 h @ 10 mA cm^{-2}	0.5 M H_2SO_4	No	Nat. Nanotechnol. 16, 1371–1377 (2021)
**$Ta_{0.1}Tm_{0.2}Ir_{0.7}O_x$**					1.451	66.4	3126 @ 1.5					
**$Tm_{0.1}Ir_{0.9}O_x$**					1.464	64.3		81.9				
**$Ta_{0.2}Ir_{0.8}O_x$**					1.47	65.7						

**$Ta_{0.1}Ir_{0.9}O_x$**				1.462	70.3		85.7				
**$Ta_{0.05}Ir_{0.95}O_x$**				1.466	68.5						
**$RuIr_3O_x$**		0.15		1.478	110.2	200 A g^{-1}_{Ir+Ru}			0.5 M H_2SO_4	No	Adv. Energy Mater.2021, 11, 2102883
**$RuIr_3O_x$**				1.478	110.2	200 A g^{-1}_{Ir+Ru}					
**$RuIrO_x$**		0.15		1.434	71.3	1225 A g^{-1}_{Ir+Ru}		110 h @ 10 mA cm^{-2}	0.5 M H_2SO_4	No	Adv. Energy Mater.2021, 11, 2102883
**Ru_3IrO_x**				1.461	93.2	350 A g^{-1}_{Ir+Ru}					
**IrO_x@$IrTiO_x$**	Ir^{3+}		0.0092	1.583	57	2622 @ 1.6 V_{RHE}		24 @ 1 mA cm^{-2}	0.5 M H_2SO_4	No	Adv. Mater. Interfaces 2022, 9, 2102035
**$Ir_{0.8}Mn_{0.2}O_x$**				1.46	42						
**$Ir_{0.7}Mn_{0.3}O_x$**				1.455	43						
**$Ir_{0.6}Mn_{0.4}O_x$**	Ir^{3+}, Ir^{4+}	0.36		1.442	43			8 h @ 10 mA cm^{-2}	0.5 M H_2SO_4	No	Int. J. Hydrog. Energy 48, 28, 2023, 10532-10544
**$Ir_{0.5}Mn_{0.5}O_x$**				1.46	46						
**$Ir_{0.4}Mn_{0.6}O_x$**				1.51	70						
**$W_{0.8}Ir_{0.2}O_y$**				1.52	51.68	900	36				
**$W_{0.7}Ir_{0.3}O_y$**	Ir^{3+}, Ir^{4+}	0.255		1.508	45.99	714.1	41	55 h @ 10 mA cm^{-2}	0.1 M $HClO_4$	Yes	ACS Appl. Mater. Interfaces 2023, 15, 6912−6922
**$W_{0.6}Ir_{0.4}O_y$**				1.53	56.61	450	63				
**$W_{0.5}Ir_{0.5}O_y$**				1.54	65.1	300	50				
**α-H_xIrO_3**		0.255		1.54	59			12 h @ 1.6 V	0.5 M H_2SO_4	No	ACS Appl. Mater. Interfaces 2023, 15, 12, 15269–15278
**β-H_xIrO_3**				1.57	76						
**Li-IrO_x**		0.286		1.62	39			2 h @ 10 mA cm^{-2}	0.1 M $HClO_4$	No	J. Am. Chem. Soc. 2023, 145, 6398−6409
**r-IrO_x**				1.52	57						

Table S2. OER performance of Ir-based mixed oxides tested in PEMWE.

Catalyst	Structure	Ir oxidation state	Mixed oxide loading mg cm^{-2}	Counter electrode	Counter electrode loading mg cm^{-2}	Water flow rate L h^{-1}	PTL	Nafion	Area cm^2	Temp. ºC	E V @ 2 A cm^{-2}	*j* A cm^{-2} @ 2 V	i_M A g^{-1}_{Cat} @ 2 V	Durability tests	References
**Sr_2CaIrO_6**	Perovskite	Ir^{5+}, Ir^{6+}	0.4	Pt	0.4	2.5	Ti porous sintered layer on Ti mesh coated with Pt	212	4	80	1.81	3.2	8000	450 h @ 2000 mA cm^{-2}	Nature Communications (2022) 13:7935
**$W_{0.7}Ir_{0.3}O_y$**		Ir^{3+}, Ir^{4+}	2	Pt/C	0.5	3	Titanium fiber felt and carbon paper were used as anode and cathode gas diffusion layers (GDL), respectively	212	5	60/ 80	2,27 /2,16	≈ 0,7	3563	90 h @ 500 mA cm^{-2}	ACS Appl. Mater. Interfaces 2023, 15, 5, 6912–6922
**$Y_2Ru_{1.2}Ir_{0.8}O_7$**	Pyrochlore		4	Pt/C	0.5		Titanium mesh and carbon paper were used as anode and cathode gas diffusion layers (GDL), respectively	117	4	Room Temperature				150 h @ 100 mA cm^{-2}	Small 2022, 18, 2202513

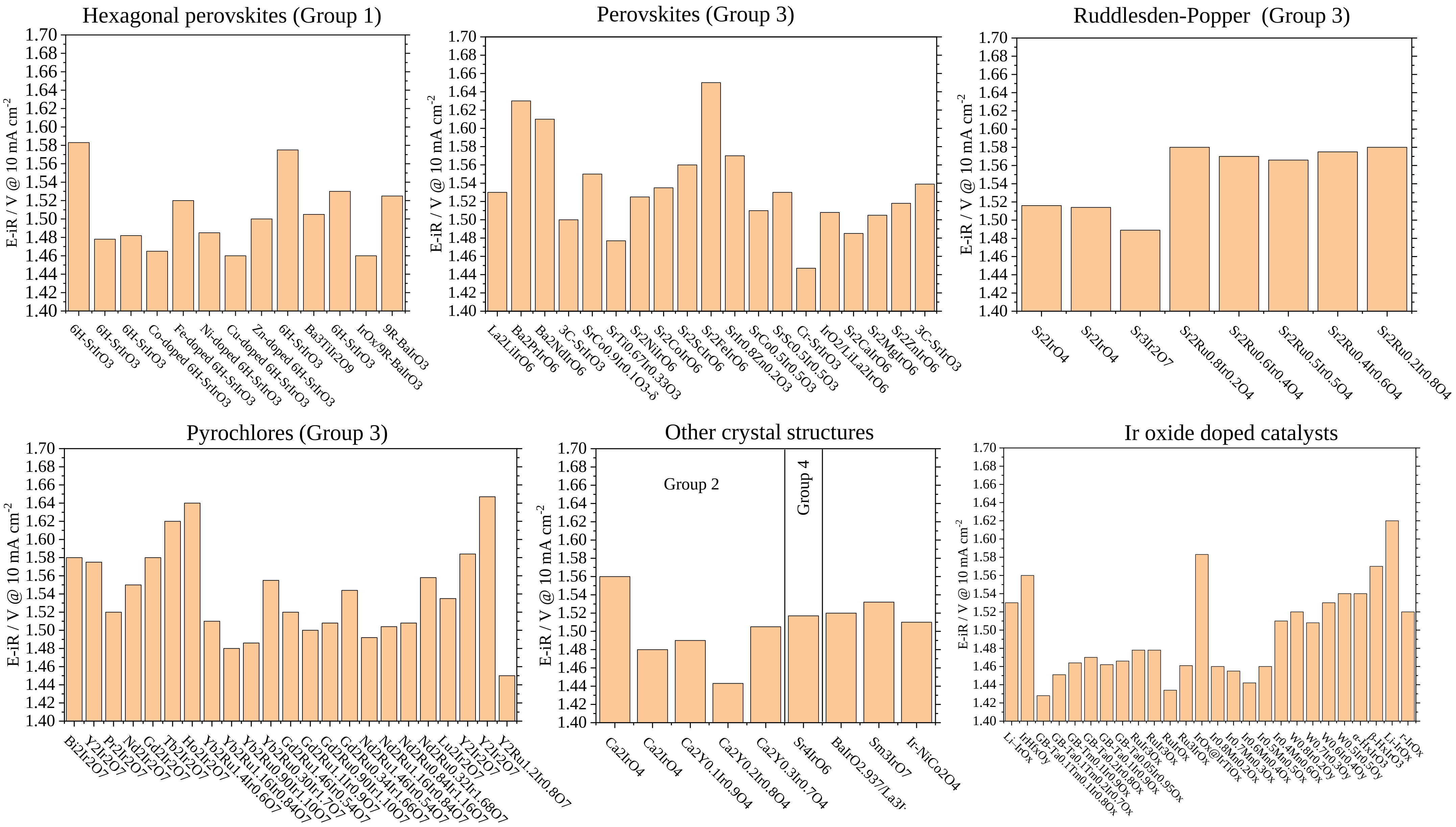

Figure S1. E-iR / V @ 10 mA cm^{-2} for Ir-based mixed oxides of group 1 (hexagonal perovskites), group 2, group 3 (perovskites, pyrochlores, Ruddlesden-Popper), group 4 and with other crystal structures in RDE. The E-iR / V @ 10 mA cm^{-2} is also shown for other Ir doped catalysts.

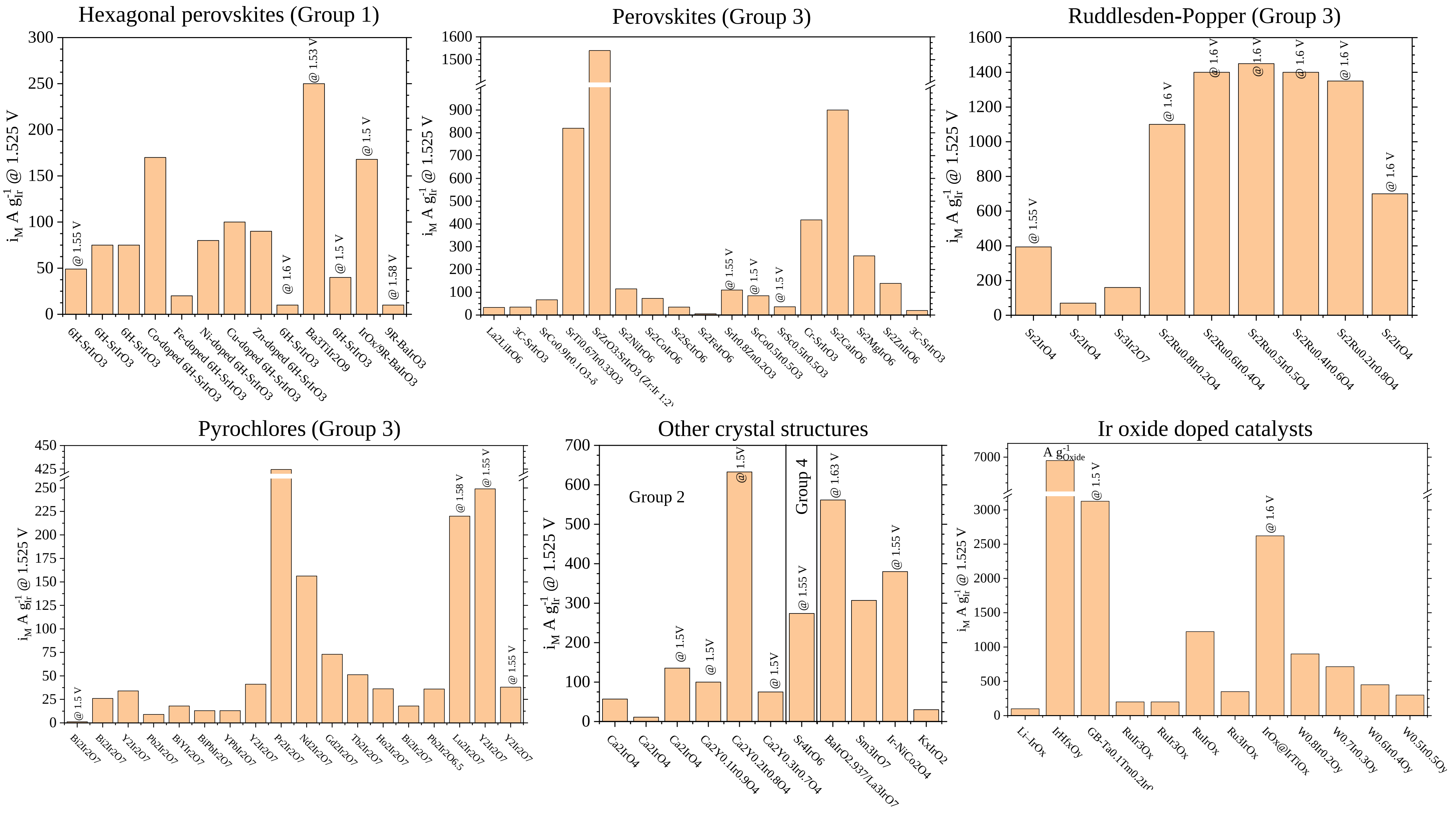

Figure S2. i_M A g^{-1}_{Ir} @ 1.525 V for Ir-based mixed oxides of group 1 (hexagonal perovskites), group 2, group 3 (perovskites, pyrochlores, Ruddlesden-Popper), group 4 and with other crystal structures in RDE. i_M A g^{-1}_{Ir} @ 1.525 V for Ir doped catalysts are also shown.

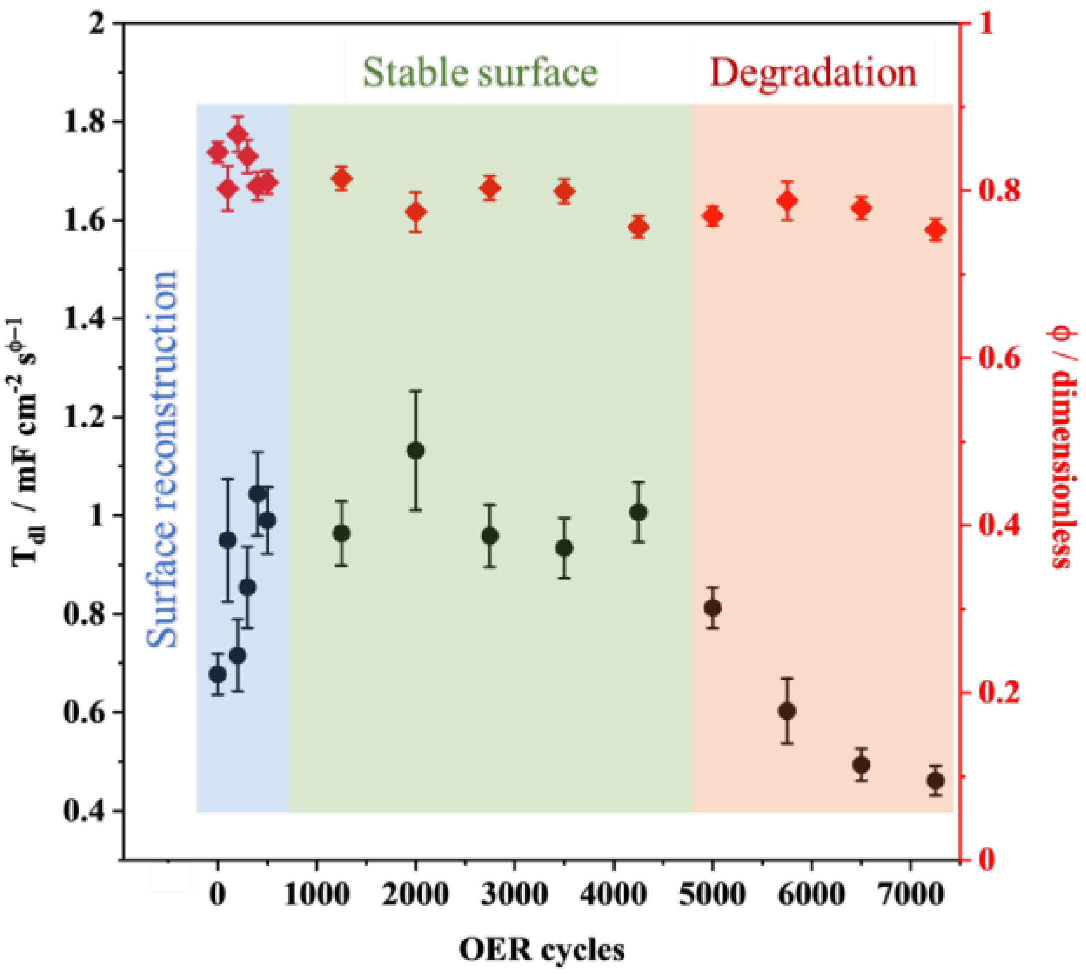

Figure S3. Monitoring of the double-layer capacitance and φ over the OER cycles performed with a Y_2MnRuO_7. The double-layer capacitance was estimated from EIS data. Three regions were identified: surface reconstruction (blue), stable surface (green) and degradation (orange) in which the double-layer capacitance increases, remains stable and decreases, respectively. Figure reprinted from *Nat. Commun.* **2023**, *14* (1), 2010, Copyright © 2023, The Author(s).
